# Perturbed myoepithelial cell differentiation in *BRCA* mutation carriers and in ductal carcinoma in situ

**DOI:** 10.1038/s41467-019-12125-5

**Published:** 2019-09-13

**Authors:** Lina Ding, Ying Su, Anne Fassl, Kunihiko Hinohara, Xintao Qiu, Nicholas W. Harper, Sung Jin Huh, Noga Bloushtain-Qimron, Bojana Jovanović, Muhammad Ekram, Xiaoyuan Zi, William C. Hines, Maša Alečković, Carlos Gil del Alcazar, Ryan J. Caulfield, Dennis M. Bonal, Quang-De Nguyen, Vanessa F. Merino, Sibgat Choudhury, Gabrielle Ethington, Laura Panos, Michael Grant, William Herlihy, Alfred Au, Gedge D. Rosson, Pedram Argani, Andrea L. Richardson, Deborah Dillon, D. Craig Allred, Kirsten Babski, Elizabeth Min Hui Kim, Charles H. McDonnell, Jon Wagner, Ron Rowberry, Kristie Bobolis, Celina G. Kleer, E. Shelley Hwang, Joanne L. Blum, Simona Cristea, Piotr Sicinski, Rong Fan, Henry W. Long, Saraswati Sukumar, So Yeon Park, Judy E. Garber, Mina Bissell, Jun Yao, Kornelia Polyak

**Affiliations:** 1 Department of Medical Oncology, Dana-Farber Cancer Institute Boston, Boston, MA 02215 USA; 2 Department of Medicine, Brigham and Women’s Hospital, Boston, MA 02115 USA; 3 Department of Medicine, Harvard Medical School, Boston, MA 02115 USA; 4 Department of Cancer Biology, Dana-Farber Cancer Institute Boston, Boston, MA 02215 USA; 5 Department of Genetics, Blavatnik Institute, Harvard Medical School, Boston, MA 02115 USA; 6 Center for Functional Cancer Epigenetics, Dana-Farber Cancer Institute, Boston, MA USA; 7 Department of Biomedical Engineering, Yale University, New Haven, CT 06511 USA; 8 Second Military Medical University, Shanghai, 200433 P.R. China; 9 Lawrence Berkeley National Laboratory, Berkeley, CA 94720 USA; 10 Lurie Family Imaging Center, Center for Biomedical Imaging in Oncology, Dana-Farber Cancer Institute Boston, Boston, MA 02215 USA; 11 Johns Hopkins University School of Medicine, Baltimore, MD 21231 USA; 12 Baylor-Charles A. Sammons Cancer Center, Dallas, TX 75246 USA; 13 University of California San Francisco Helen Diller Family Comprehensive Cancer Center, San Francisco, CA 94143 USA; 14 Department of Pathology, Brigham and Women’s Hospital, Boston, MA 02115 USA; 15 Department of Pathology, Harvard Medical School, Boston, MA 02115 USA; 16 Department of Pathology, Washington University School of Medicine, St. Louis, MO 63110 USA; 17 Sutter Roseville Medical Center, Roseville, CA 95661 USA; 18 Department of Pathology, University of Michigan, Ann Arbor, MI 48109 USA; 19 Department of Data Science, Dana-Farber Cancer Institute Boston, Boston, MA 02215 USA; 20 Department of Biostatistics, Harvard T. H. Chan School of Public Health Boston, Boston, MA 02215 USA; 21 Department of Stem Cell and Regenerative Biology, Harvard University Cambridge, Cambridge, MA 02138 USA; 22 Department of Pathology, Seoul National University College of Medicine, Seoul, Korea; 23 MD Anderson Cancer Center, Houston, TX 77030 USA; 24 Harvard Stem Cell Institute, Cambridge, MA 02138 USA; 25 Present Address: Deciphera Pharmaceuticals, Waltham, MA USA; 26 Present Address: Nagoya University Graduate School of Medicine, Nagoya, Japan; 27 Present Address: ImmunoGen, Inc, Waltham, MA USA; 28 Present Address: EMEA Site Intelligence and Activation, Tel Aviv, Israel; 29 Present Address: WuXi NextCODE, Cambridge, MA USA; 30 Present Address: University of New Mexico School of Medicine, Albuquerque, NM USA; 31 Present Address: Metamark Genetics Inc, Worcester, MA USA; 32 Present Address: Johns Hopkins Medical Institutions, Baltimore, MD USA; 33 Present Address: Cancer Treatment Centers of America, Atlanta, GA USA; 34 Present Address: Duke University, Durham, NC USA

**Keywords:** Breast cancer, Transcriptomics

## Abstract

Myoepithelial cells play key roles in normal mammary gland development and in limiting pre-invasive to invasive breast tumor progression, yet their differentiation and perturbation in ductal carcinoma in situ (DCIS) are poorly understood. Here, we investigated myoepithelial cells in normal breast tissues of *BRCA1* and *BRCA2* germline mutation carriers and in non-carrier controls, and in sporadic DCIS. We found that in the normal breast of non-carriers, myoepithelial cells frequently co-express the p63 and TCF7 transcription factors and that p63 and TCF7 show overlapping chromatin peaks associated with differentiated myoepithelium-specific genes. In contrast, in normal breast tissues of *BRCA1* mutation carriers the frequency of p63^+^TCF7^+^ myoepithelial cells is significantly decreased and p63 and TCF7 chromatin peaks do not overlap. These myoepithelial perturbations in normal breast tissues of *BRCA1* germline mutation carriers may play a role in their higher risk of breast cancer. The fraction of p63^+^TCF7^+^ myoepithelial cells is also significantly decreased in DCIS, which may be associated with invasive progression.

## Abstract (web summary)

Myoepithelial cells prevent tumour growth and invasion in DCIS. Here, the authors show that p63 and TCF7 cooperate to regulate a transcription factor network for the maintenance of normal myoepithelial function and altered expression of these genes perturb myoepithelial function in DCIS to promote invasive progression.

## Introduction

The normal mammary gland is composed of multiple cell types including luminal and myoepithelial cells^1–3^. Myoepithelial cells produce and are in direct contact with the basement membrane (BM). Together, these cells outline the inner luminal epithelial cells in both ducts and alveoli, serving as a structural barrier, and they regulate luminal epithelial cell differentiation and polarity^1–3^. Upon lactation and breastfeeding, myoepithelial cells contract hence propelling milk out of the lumens in response to oxytocin. Normal myoepithelial cell differentiation is still poorly understood, although Notch, TGFβ, Hh, and HER1-ERK1/2-RSK signaling pathways, and the p63 transcription factor (TF) have been implicated^4–9^.

Myoepithelial cells also inhibit neoplastic phenotypes including tumor cell growth, invasion, and angiogenesis^10–13^. The presence or absence of an intact myoepithelial cell layer and BM, assessed based on histology and by immunohistochemical analyses for myoepithelial markers including CD10, p63, and SMA, differentiat in situ from invasive breast carcinomas^14^. Immunohistochemical analyses of normal breast tissue, and in situ and invasive breast carcinomas aiming to identify markers of tumor progression have identified several genes that are differentially expressed between normal and DCIS-associated myoepithelial cells including SMMHC, CK5/6, CD10, calponin, and integrin αvβ6^15–21^. Decreased expression of CD10 (*MME*) was also reported to predict recurrence in patients with DCIS, but the number of cases analyzed was small and the patients received variable treatments^22^. Similarly, loss of myoepithelial CD10 expression by immunohistochemical staining was associated with stromal invasion^23^. Myoepithelial CD10 expression may have functional relevance, since it is a membrane-associated zinc-dependent neutral endopeptidase that can cleave glucagon, enkephalins, and oxytocin^24^. In normal mouse mammary gland oxytocin enhances myoepithelial cell proliferation and differentiation^25^, while in cell culture CD10 inhibition decreases proliferation^26^. Thus, the decreased CD10 expression in DCIS-associated myoepithelial cells may contribute to their progressive loss^27^.

We previously purified myoepithelial cells using the CD10 cell surface marker from normal reduction mammoplasties and DCIS, and determined that their DNA methylation and gene expression patterns are distinct implying malignancy-associated changes in DCIS^28,29^. We also showed that myoepithelial cells prevent tumor growth and invasive progression in experimental models of DCIS^6,30^, suggesting that perturbed myoepithelial cell function in DCIS may be permissive for tumor progression. DCIS-associated myoepithelial cells also overexpress immune-regulatory proteins (e.g., CXCL12, CXCL14, and PD-L1)^28,31^ suggesting a role in immune evasion. Thus, loss of normal myoepithelial cells in DCIS might be key for in situ to invasive breast carcinoma transition and it may identify patients with high risk of progression. However, the regulators of normal myoepithelial differentiation programs and how these may be altered in DCIS are not well understood.

Here we used a combination of genomic profiling of human normal and DCIS breast tissues and functional assays in the MCF10DCIS experimental model of DCIS^32^ to investigate determinants of normal myoepithelial cell differentiation and perturbations of these in *BRCA1* and *BRCA2* germline mutation carriers and in DCIS. Luminal differentiation was shown to be perturbed in *BRCA1* mutation carriers^33–35^, but myoepithelial cells and *BRCA2* mutation carriers have not been investigated. We defined the genomic targets of p63 and TCF7, two TFs we identified as co-expressed in the majority of myoepithelial cells in normal breast tissue of non-carrier women but not in *BRCA1/2* mutation carriers and in DCIS, and the enhancer landscape in normal myoepithelial cells. We also characterized the functional relevance of p63 and TCF7 co-expression and their targets in MCF10DCIS cells. Our results suggest that a transcriptional program orchestrated by p63 and TCF7 is required for a normal differentiated myoepithelial cell phenotype and perturbations of this may contribute to the increased breast cancer risk of *BRCA* mutation carriers, and it may lead to the loss of myoepithelial cells in DCIS promoting progression to invasion.

## Results

### Heterogeneity of normal CD10^+^ myoepithelial cell population

**Fig. 1:**
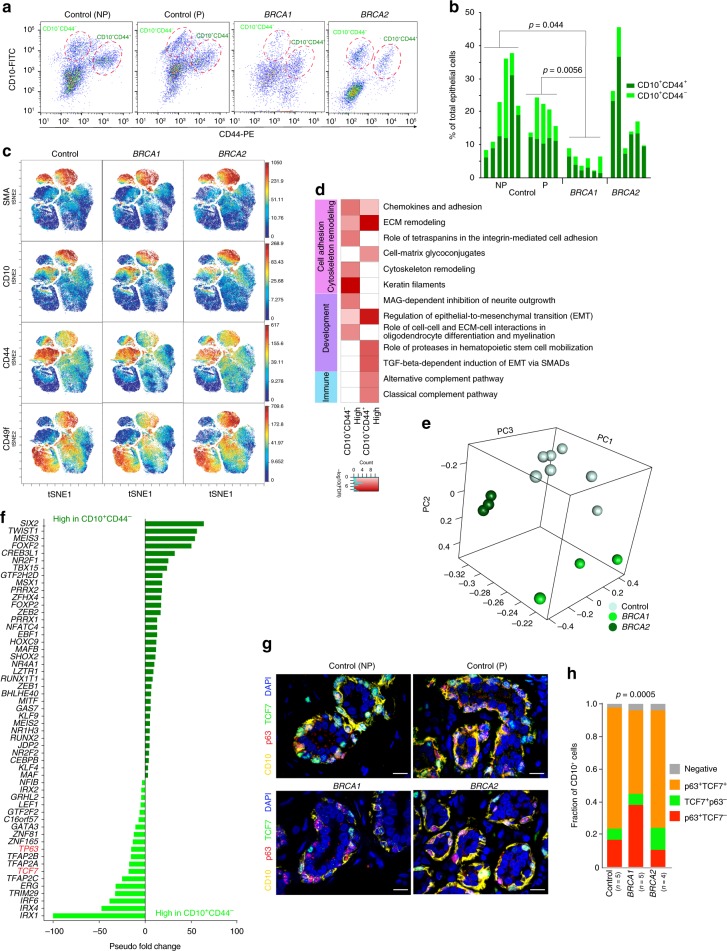
Heterogeneity of the CD10^+^ cell population. **a** FACS analysis of CD10^+^ cells according to expression of CD44 in normal breast tissues of nulliparous (NP) and parous (P) control women and *BRCA1* and *BRCA2* mutation carriers. **b** Quantification of percentage of CD10^+^CD44^−^ and CD10^+^CD44^+^ in total epithelial cells (*n* = 6/group). *p*-values indicate statistical significance of difference in total CD10^+^ cells between groups by *t*-test. **c** viSNE maps from CyTOF analysis of normal breast tissues colored for expression of SMA, CD10, CD44, and CD49f (control *n* = 6, *BRCA1* *n* = 6, *BRCA2* *n* = 7). Color scale indicates minimum and maximum values of expression. **d** Pathways enriched in genes differentially expressed between CD10^+^CD44^−^ and CD10^+^CD44^+^ cells. Color scale corresponds to −log(*p*-value) of significance of enrichment, calculated by MetaCore Enrichment Analysis test. **e** 3D Principal component analysis plots of CD10^+^ gene expression data from the indicated samples. **f** Transcription factors differentially expressed between CD10^+^CD44^−^ and CD10^+^CD44^+^ cells. Red highlight indicates genes selected for further analyses. **g** Multicolor immunofluorescence analysis of p63, TCF7, and CD10 expression in normal breast tissues. Images are a montage of nine fields captured from one area of the tissue. Scale bar 50 μm. **h** Relative quantification of CD10^+^ myoepithelial cells positive for p63 or TCF7 or both proteins. *p*-value indicates the significant association of the p63/TCF7 positive/negative status of CD10^+^ myoepithelial cells with condition (Control, *BRCA1*, or *BRCA2*), as assessed by Pearson’s chi-squared test among averages of estimated cell counts across replicates (total population size was conservatively estimated to 100 cells). Source data are provided as a Source Data file

CD10 is a myoepithelial cell surface marker and its expression level may vary depending on differentiation state^22,23,26^, thus we explored CD10^+^ cell population heterogeneity in normal human breast tissues by multicolor FACS for CD10 and markers known to be associated with basal/progenitor features including CD44, ITGA3, ITGB6, and ITGA6^6,28,36–39^. We analyzed normal breast tissues of nulliparous and parous women, as pregnancy and lactation may impact cellular phenotypes^40^, from reduction mammoplasties and from prophylactic mastectomy tissues of *BRCA1* and *BRCA2* mutation carriers (Supplementary Data 1). Women were as closely matched as possible for menopausal status, ethnicity, and age. We identified two distinct CD10^+^ cell populations distinguished by the expression of CD44 that were both CK14^+^, but CD10^+^CD44^+^ cells were more mesenchymal and CD10^+^CD44^−^ cells more epithelial (Fig. 1a and Supplementary Fig. 1a). We also assessed ALDH activity, a feature of stem/progenitor cells^41^, in three distinct CD10^+^ cell subpopulations (i.e., CD10^high^CD44^−^, CD10^low^CD44^−^, and CD10^+^CD44^+^). ALDH^+^ cells were mainly present in the CD10^+^CD44^+^ subset, where ~37% of the cells displayed ALDH activity suggesting the presence of progenitors (Supplementary Fig. 1b).

Quantification of the relative fraction of total CD10^+^ cells and CD10^+^CD44^−^ and CD10^+^CD44^+^ subpopulations demonstrated a significant decrease in CD10^+^ cells in *BRCA1* mutation carriers (Fig. 1b and Supplementary Fig. 1c). CyTOF analysis of non-carrier (*n* = 6), *BRCA1* (*n* = 6), and *BRCA2* (*n* = 7) samples using myoepithelial, luminal, basal, and progenitor markers also demonstrated significant differences with a decrease in SMA^+^CD10^+^ myoepithelial cells and diminished expression of SMA, CD10, CD44, and CD49f in *BRCA1* mutation carriers (Fig. 1c and Supplementary Fig. 1d, e). To minimize individual or age-related differences, all samples for a respective mutation status (i.e., control, *BRCA1*, *BRCA2*) were concatenated and used as a single file for analysis. These data suggest that the phenotype of myoepithelial cells is distinct between normal tissues of non-carrier and *BRCA* mutation-carrier women.

### Gene expression profiles of CD10^+^ cell populations

Next, we analyzed CD10^+^CD44^−^ and CD10^+^CD44^+^ cell gene expression profiles from reduction mammoplasty samples (*n* = 3 each, from nulliparous and parous women). Known myoepithelial cell markers (e.g., *ACTG2*, *SFN*, and *OXTR*) had higher expression in CD10^+^CD44^−^ cells, while stem/progenitor cell markers (e.g., *ALDH1A1*, *WNT2*, and *KLF4*), and epithelial-to-mesenchymal transition (EMT)-related genes (e.g., *TWIST1* and *ZEB1*) were more abundantly expressed in CD10^+^CD44^+^ cells (Supplementary Fig. 1f and Supplementary Data 2). Pathway analysis using Metacore^42^ revealed enrichment for extracellular matrix (ECM) remodeling and TGFβ signaling in CD10^+^CD44^+^ cells, whereas keratin, chemokines, and adhesion pathways were more enriched in genes highly expressed in CD10^+^CD44^−^ cells (Fig. 1d). Parity-related differences were limited and enriched in ECM remodeling pathways (Supplementary Fig. 1g).

Next, we profiled CD10^+^ cells from *BRCA1* and *BRCA2* mutation carriers and compared them to non-carriers. Principal component analysis (PCA) depicted three distinct groups reflecting germline mutation status (Fig. 1e). Genes highly expressed in *BRCA1*-mutant CD10^+^ cells were enriched in DNA replication-related functions, whereas *BRCA2*-mutant CD10^+^ cells showed a decrease in keratins and an increase in immune-related genes (Supplementary Fig. 1g and Supplementary Data 3).

TFs play key roles in cellular differentiation. Thus, we identified TFs differentially expressed between CD10^+^CD44^−^ and CD10^+^CD44^+^ cells including many homeobox genes (e.g., *IRX1*, *IRX4*, and *IRX2*) and genes with known roles in epithelial differentiation (e.g., *GATA3* and *TFAP2A*) and in myoepithelial cells (e.g., *TP63*) (Fig. 1f). Among these TFs, *TP63* and *TCF7* were particularly interesting, since p63 plays key roles in epithelial progenitors^43,44^, whereas TCF7 regulates WNT signaling and its deletion in mice leads to mammary gland adenomas^45^. Both *TP63* and *TCF7* have multiple functionally distinct isoforms^46,47^. Based on RNA-seq we detected ΔNp63 and the long isoform of TCF7 in normal myoepithelial cells (Supplementary Fig. 1h, i).

We further analyzed the expression of p63 and TCF7 in normal breast tissues by multicolor immunofluorescence in a larger cohort. We found that in control non-carrier women and in *BRCA2* mutation carriers the majority of CD10^+^ myoepithelial cells were p63^+^TCF7^+^, but in *BRCA1* mutation carriers the expression of p63 and TCF7 decreased, and co-localization was less frequent (Fig. 1g, h). These data indicate altered myoepithelial cell phenotypes in *BRCA1* mutation carriers and suggest key roles for p63 and TCF7 in normal myoepithelial cell differentiation.

### Myoepithelial cells in DCIS

**Fig. 2:**
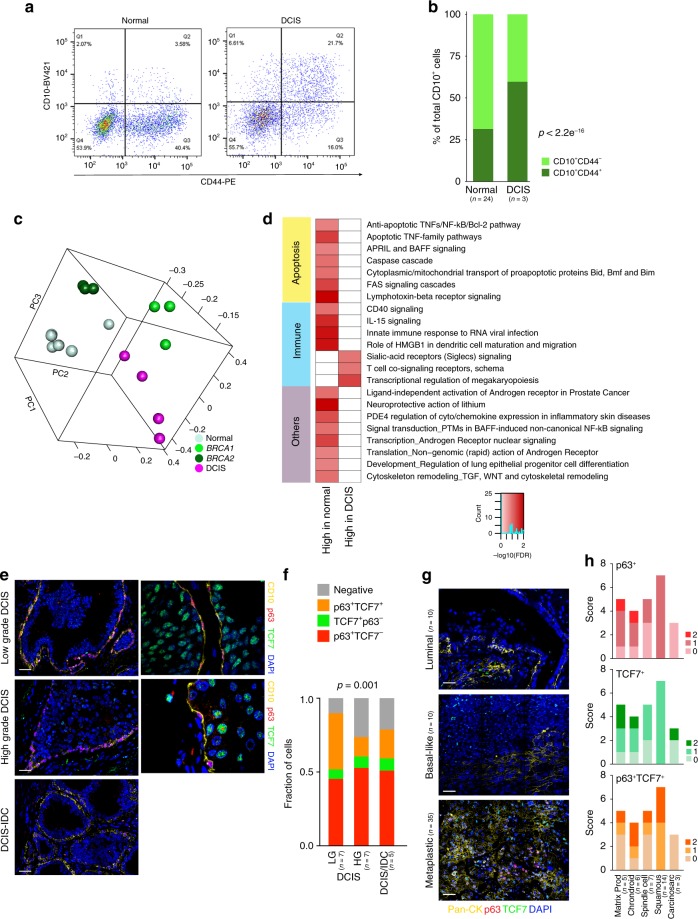
CD10, p63, and TCF7 expression in DCIS and in invasive breast tumors. **a** FACS analysis of CD10 and CD44 expression in DCIS and in normal breast tissue. **b** Relative quantification of CD10^+^CD44^−^ and CD10^+^CD44^+^ in total epithelial cells. *p*-value indicates the significant association of the CD44 positive/negative status of CD10^+^ cells with condition (Normal, DCIS), as assessed by Pearson’s chi-squared test among averages of estimated cell counts across replicates (total population size was conservatively estimated to 100 cells). **c** 3D principal component analysis plot of gene expression data. **d** Pathways enriched in genes differentially expressed between CD10^+^ cells in DCIS and in normal breast. Color scale corresponds to −log(*p*-value) of significance of enrichment, calculated by MetaCore Enrichment Analysis test. **e** Multicolor immunofluorescence analysis of p63, TCF7, and CD10 expression in low grade (LG) and high grade (HG) DCIS and DCIS-IDC. Left panels are a montage of nine fields captured from one area of the tissue, while right panels are high magnification of selected areas. Scale bar 50 μm. **f** Relative quantification of CD10^+^ cells positive for p63 or TCF7 or both proteins in low (LG) and high (HG) grade pure DCIS and DCIS adjacent to IDC (DCIS-IDC). *p*-value indicates the significant association of the p63/TCF7 positive/negative status of CD10^+^ cells with condition (LG-DCIS, HG-DCIS, and DCIS-IDC), as assessed by Pearson’s chi-squared test among averages of estimated cell counts across replicates (total population size was conservatively estimated to 100 cells). **g** Multicolor immunofluorescence analysis of PanCK, p63, and TCF7 in luminal, basal-like, and metaplastic invasive breast tumors. Images are a montage of nine fields captured from one area of the tissue. Scale bar 50 μm. **h** Scoring the expression and co-localization of p63 and TCF7 in different subtypes of metaplastic breast tumors. Source data are provided as a Source Data file

In DCIS, cancer cells are growing within normal mammary ducts, thus, tumor epithelial and surrounding myoepithelial cells thought to be clonally unrelated. To investigate CD10^+^ myoepithelial cells in DCIS we first analyzed the ratios of CD10^+^CD44^−^ and CD10^+^CD44^+^ cells by FACS. We found that in DCIS the majority of CD10^+^ cells were also CD44^+^ (Fig. 2a, b) and the gene expression profiles of CD10^+^ cells from DCIS were distinct from CD10^+^ cells in normal breast tissues (Fig. 2c, Supplementary Fig. 2a, and Supplementary Data 4). Pathway analysis showed enrichment for several immune-related signaling pathways in both DCIS-high and normal-high genes (Fig. 2d), implying a role in immune regulation.

We also performed immunofluorescence analysis of p63 and TCF7 expression in pure DCIS, DCIS adjacent to IDC, and IDC. There was a striking loss of TCF7 levels in CD10^+^ cells in DCIS, especially in high-grade lesions, leading to a significant decrease in p63^+^TCF7^+^ cells compared to normal breast (Fig. 2e, f). Instead, a subset of DCIS tumor epithelial cells was TCF7^+^. To assess if this tumor epithelial expression of TCF7 is maintained during invasive progression, we also analyzed invasive tumors of different subtypes. Luminal and basal-like tumors had a nearly complete lack of p63 and TCF7 expression, although a subset of leukocytes was TCF7^+^ (Fig. 2g). In contrast, in metaplastic breast cancer a variable fraction of cancer cells expressed p63 or TCF7 or both proteins with squamous tumors having the highest frequency of p63^+^TCF7^+^ cells (Fig. 2g, h). Metaplastic breast tumors are the only breast cancer subset that relatively commonly have mutations in the APC/β-catenin pathway^48^ and the expression of TCF7 could be due to its induction by WNT/β-catenin signaling^45^. Overall, these data demonstrate perturbed myoepithelial cell differentiation in DCIS associated with altered cellular expression of TCF7.

### Targets of p63 and TCF7 and normal myoepithelial enhancers

**Fig. 3:**
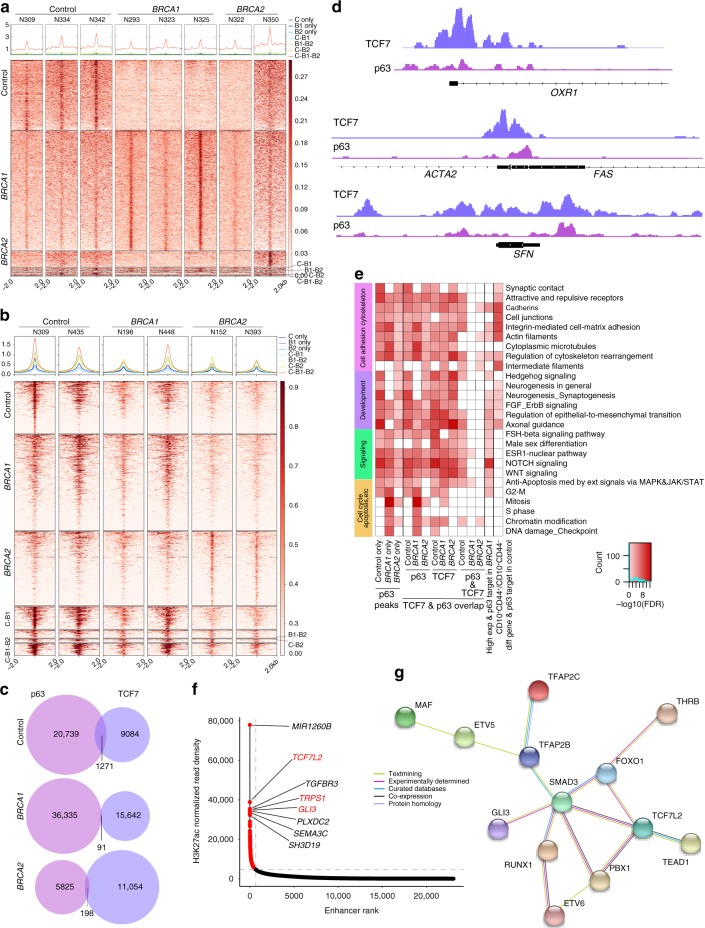
p63 genomic targets of and enhancer landscape in myoepithelial cells. **a**, **b** Heatmap depicting p63 (**a**) and TCF7 (**b**) peaks which are unique in normal breast tissue of control women (C only), and *BRCA1* (B1 only), and *BRCA2* (B2 only) mutation carriers, and overlap between groups. The color key is the score of ChIP-seq signal over selected genomic region, the signals across different genomic regions have scaled to the same length. **c** Overlap between p63 peaks and TCF7 peaks in control women, *BRCA1* and *BRCA2* mutation carriers. **d** Gene tracks depicting p63 and TCF7 signal at selected genomic loci. The *x*-axis shows position along the chromosome with gene structures drawn below, whereas *y*-axis shows genomic occupancy in units of rpm/bp. **e** Pathway-enrichment analysis of genes associated with p63 or TCF7 peaks in normal, *BRCA1*, or *BRCA2* myoepithelial cells or differentially expressed between the indicated cell types. Color scale corresponds to −log(*p*-value) of significance of enrichment, calculated by MetaCore enrichment analysis test. **f** Plot depicting super-enhancers in normal myoepithelial cells from control women. **g** Predicted protein interaction network of TFs identified as core transcriptional regulatory circuits in myoepithelial cells. Legend indicates the source of data used to determine interactions. TFs that are not part of the network were removed

Next, we investigated the genomic targets of p63 and TCF7 and potential differences due to *BRCA* mutation status in normal myoepithelial cells by ChIP-seq. We identified significant differences in both p63 and TCF7 genomic binding between control and *BRCA* mutation carriers (Fig. 3a, b, Supplementary Fig. 2b, and Supplementary Data 5). We detected significant overlap between p63 and TCF7 peaks only in non-carrier tissues and not in *BRCA1/2* mutation carriers consistent with the decreased frequencies of p63^+^TCF7^+^ cells in these tissues (Fig. 3c). Interestingly, normal myoepithelial cell-specific genes including *ACTA2*, *SFN*, and *OXR1* were associated with overlapping p63 and TCF7 peaks suggesting that the co-localization of these two TFs may regulate their expression (Fig. 3d). Metacore analysis of p63 and TCF7 targets revealed that most of the top pathways were commonly enriched in all three tissue types including WNT, Hh, and NOTCH, cell adhesion, and cell–matrix interaction signaling (Fig. 3e), consistent with the role of p63 in epithelial stem cells and cell adhesion^49^. Mitosis and DNA damage checkpoint pathways were more significantly enriched in *BRCA1* mutation carriers potentially due to DNA repair defects even in *BRCA1*^*+/−*^ cells (Fig. 3e). TCF7 peaks were enriched in the same process networks as p63, while p63 and TCF7 overlapping peaks showed less significant enrichment and in fewer networks.

Integration of p63 ChIP-seq data with differentially expressed gene lists in CD10^+^ cells showed enrichment for NOTCH and WNT signaling in p63 targets with higher expression in *BRCA1* tissues implying more progenitor-like features (Fig. 3e). The majority of genes with higher expression in CD10^+^CD44^−^ cells were also direct p63 targets, and many were related to cell adhesion, cytoskeleton, and neurogenesis-axonal guidance confirming a central role for p63 in these cells (Fig. 3e).

Enhancers and super-enhancers (SE) play important roles in establishing cellular identity^50^. Thus, we also characterized the enhancer landscape of normal CD10^+^ myoepithelial cells by ChIP-seq for histone H3 lysine 27 acetyl (H3K27ac). We identified 671 myoepithelial cell SEs that included several TFs (e.g., *TCF7L2*, *TRPS1*, *GLI3*), genes encoding axonal guidance (e.g., *SEMA3C, SEMA5A*) and cytoskeletal proteins (e.g., *PALLD, TNS1*), TGFβ-signaling pathway components (e.g., *TGFBR3, SMAD3*), and genes involved in muscle differentiation (e.g., *BOC, MYLK, CALD1*) (Fig. 3f and Supplementary Data 6). We also subject the SE data to core transcriptional circuitry (CRC) analysis^51^ and identified 14 TFs predicted to be core regulators of myoepithelial cell state. To analyze interaction networks among these 14 TFs, we performed protein–protein interactome analysis using the STRING database^52^ as described in Supplementary Methods. 13 out of 14 TFs formed a tight interaction network with SMAD3 and TCF7L2 as major hubs (Fig. 3g), suggesting that these TFs are the master regulators of the myoepithelial cell SE landscape and phenotype.

### Functional relevance of p63 in MCF10DCIS cells

**Fig. 4:**
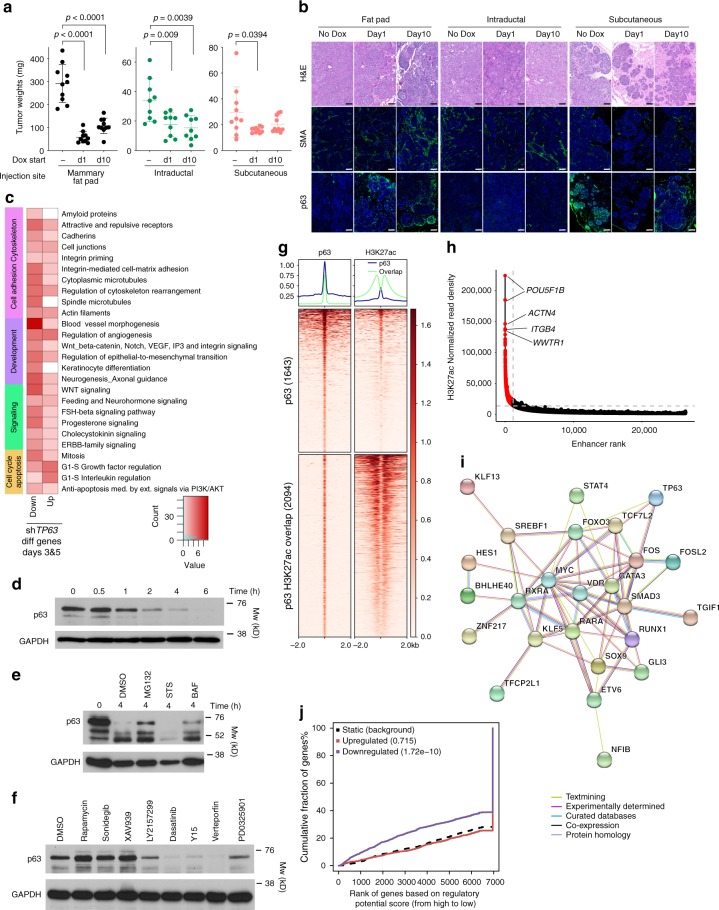
Functional relevance of p63 in myoepithelial cells. **a** Xenograft tumor weight of sh*TP63*-expressing MCF10DCIS cells with or without doxycycline following mammary fat pad, intraductal, or subcutaneous injection. *p*-value indicates statistical significance of difference in tumor weight between groups based on *t*-test. Mean ± SD shown. **b** Hematoxylin–eosin (H&E) staining and immunofluorescence analysis of SMA and p63 expression. Scale bar 100 μm. **c** Pathway enrichment analysis of genes up-regulated or down-regulated following sh*TP63* expression in MCF10DCIS cells. Color scale corresponds to −log(*p*-value) of significance of enrichment, calculated by MetaCore enrichment analysis test. **d**–**f** Immunoblot analysis of p63 expression levels in MCF10DCIS cells following detachment from matrix **d**, detachment and concomitant treatment with MG132 (10 µM), Staurosporin (STS, 10 µM), and Bafilomycin A1 (BAF 100 nM) **e**, and treatment with inhibitors of various signaling pathways [Rapamycin (mTOR), Y15 (FAK) 5 µM; Sonidegib (Hh), XAV393 (WNT), LY2157299 (TGFβ), Verteporfin (YAP), PD0325901 (MEK) 10 µM; Dasatinib (SRC) 2 µM] in adherent conditions **f**. GAPDH serves as loading control. **g** Heatmap depicting unique and H3K27ac overlapping p63 peaks. The color key is the score of ChIP-seq signal over selected genomic region, the signals across different genomic regions have scaled to the same length. **h** Hockey stick plot depicting super-enhancers in MCF10DCIS cells. **i** Predicted protein interaction network of TFs identified as core transcriptional regulatory circuits in MCF10DCIS cells. Legend indicates the source of data used to determine interactions. TFs that are not part of the network were removed. **j** Integration of differential gene expression and p63 targets by BETA analysis. The *p*-value listed in the top left represents the significance of the UP or DOWN group relative to the NON group as determined by the Kolmogorov–Smirnov test. Source data are provided as a Source Data file

To address the functional relevance of p63 and TCF7 in regulating myoepithelial cell features, we used the MCF10DCIS human xenograft model^6,32^ as we were not able to grow and manipulate normal CD10^+^ myoepithelial cells. MCF10DCIS cells form DCIS-like lesions with a myoepithelial cell layer at early time points after injection that progress to invasive tumors^6,32^. Thus, while MCF10DCIS cells are tumorigenic, they have the ability to differentiate into cells with myoepithelial features making them useful for the analysis of this process. We previously showed that MCF10DCIS cells express ΔNp63 and in cell culture virtually all cells are p63^+^^6^, while in DCIS-like xenografts only the myoepithelial cells remain p63^+^. We downregulated p63 using TET-inducible shRNAs (Supplementary Fig. 3a) and analyzed myoepithelial cell differentiation in xenograft assays. We performed subcutaneous, mammary fat pad, and intraductal injections to test if the microenvironment affects the phenotype of the tumors and p63 expression. MCF10DCIS cells expressing TET-inducible sh*TP63* efficiently formed tumors in NSG mice regardless of the injection site, although there were significant differences in tumor size with mammary fat pad tumors being the largest (Fig. 4a). Based on our prior studies, the establishment of DCIS-like histology requires 7–10 days after tumor initiation^6^, thus, sh*TP63* was induced 1 day or 10 days after injection and tumors were harvested 3 weeks after injection. Downregulation of p63 significantly reduced tumor weight regardless of injection site and time of sh*TP63* induction (Fig. 4a). However, while most tumors in the fat pad and intraductal groups were invasive, subcutaneous tumors were mostly DCIS-like (Fig. 4b). Based on immunofluorescence there was a nearly complete absence of SMA in myoepithelial cells in the subcutaneous tumors, while in fat pad and intraductal tumors there were many SMA^+^ myofibroblasts (Fig. 4b). Unexpectedly, myoepithelial cells of DCIS-like tumors were weakly p63^+^ in all cases except in intraductal tumors implying escape from shRNA effect (Fig. 4b). These data demonstrate that the microenvironment has a pronounced effect on p63 expression and tumor histology.

To investigate transcriptional changes after p63 downregulation, we analyzed the gene expression profiles by RNA-seq at days 3 and 5 following sh*TP63* induction (time points chosen based on assessment of p63 protein levels) (Supplementary Data 7). Pathway analysis showed an enrichment for cell cycle G2-M (e.g., cyclin B, BUB1), S phase, WNT signaling (e.g., TCF7L1, TCF3) in genes downregulated following p63 loss, whereas upregulated genes were enriched for factors involved in G1-S phase transition (Fig. 4c).

To explore the relevance of cell adhesion and other signaling pathways in regulating p63 function, we analyzed p63 protein levels at different time points after detachment from ECM. We found that p63 protein level is rapidly decreased after detachment, consistent with our prior data^6^ (Fig. 4d). To determine if the detachment-induced decline in p63 protein levels is due to increased degradation, we treated cells with proteosomal (MG132), protein kinase (Staurosporine, STS), and lysosomal (Bafilomycin A1, BAF) inhibitors. MG132 and BAF treatment significantly inhibited p63 loss, implicating proteosomal and lysosomal degradation as potential underlying mechanisms (Fig. 4e). To further investigate the regulation of p63 protein stability, we explored if inhibition of certain signaling pathways could induce a decrease in p63 protein levels even in attached cells. We found that mTOR (rapamycin), Hh (sonidegib), and WNT (XAV939) inhibitors had no effect on p63 protein levels, while treatment with TGFBR (LY2157299), MEK/ERK (PD0325901), SRC (dasatinib), FAK (Y15), and Hippo (verteporfin) inhibitors lead to a significant decrease (Fig. 4f). These data suggest that p63 regulates the expression of ECM and cell adhesion proteins and p63 protein stability is regulated by ECM attachment via SRC, FAK, and HIPPO signaling creating a positive feedback loop. However, since we generated these data in the MCF10DCIS model, these findings need to be validated in normal myoepithelial cells.

### p63 targets and enhancer landscape in MCF10DCIS cells

To investigate the genomic targets of p63 and the enhancer landscape in MCF10DCIS cells and to compare these with normal myoepithelium, we performed ChIP-seq for p63 and H3K27ac (Fig. 4g). Pathway analysis of genes nearest to p63 and H3K27ac overlapping peaks demonstrated enrichment in G1-S regulation, cadherin-mediated and integrin-mediated cell adhesion, NOTCH, and WNT signaling (Supplementary Fig. 3b). Normal myoepithelial-specific genes including *ACTA2* and several TFs highly expressed in CD10^+^CD44^−^ cells (e.g., *TCF7*, *IRF6*, *TRIM29*) were also direct targets of p63 in MCF10DCIS cells. Overall, we identified 1,233 p63 targets that were common between MCF10DCIS cells and normal myoepithelium suggesting that this model reproduces at least some aspects of normal myoepithelial cell differentiation.

Next, we performed SE analysis of the H3K27ac ChIP-seq data and identified 1,178 SEs. Top SEs included genes encoding cell adhesions proteins (e.g., *ITGA6*, *ITGB4*, *ITGB1*, *ITGB6*), keratins (e.g., *KRT5*), TFs co-expressed with p63 in normal myoepithelial cells (e.g., *GATA3*, *IRX2*, *IRX4*), and TGFβ pathway components (e.g., *TGFBR2*) (Fig. 4h and Supplementary Data 6). As expected, genes nearest to SEs showed higher gene expression levels, and we also observed a significant enrichment of p63 peaks in higher ranking SEs (Supplementary Fig. 3c), highlighting its importance in regulating basal cell-specific transcription programs. CRC^51^ analysis of SEs identified 29 top TFs that formed a tight interaction network with MYC and SMAD3, as major hubs based on the STRING database^52^ (Fig. 4i). SMAD3 was also a major hub in normal myoepithelial cells highlighting its importance in these cell types.

To further define associations between p63 chromatin binding and transcription, we integrated p63 ChIP-seq with genes differentially expressed after sh*TP63* induction and performed binding and expression target analysis (BETA)^53^. We found that genes downregulated after p63 loss were significantly enriched in p63 peaks (Fig. 4j), thus, p63 functions as a transcriptional activator in MCF10DCIS cells. Pathways analysis of downregulated genes that are direct p63 targets demonstrated enrichment in blood vessel morphogenesis, mitosis, cell cycle G2-M, NOTCH and WNT signaling, and neurogenesis-related pathways (Supplementary Fig. 3d), which is consistent with the apparent decrease in cell proliferation after sh*TP63* expression (Supplementary Fig. 4a, b). These data demonstrate that p63 is required for myoepithelial cell features and is a major regulator of the enhancer landscape in the MCF10DCIS model.

### Targets and functional relevance of TCF7 in MCF10DCIS cells

**Fig. 5:**
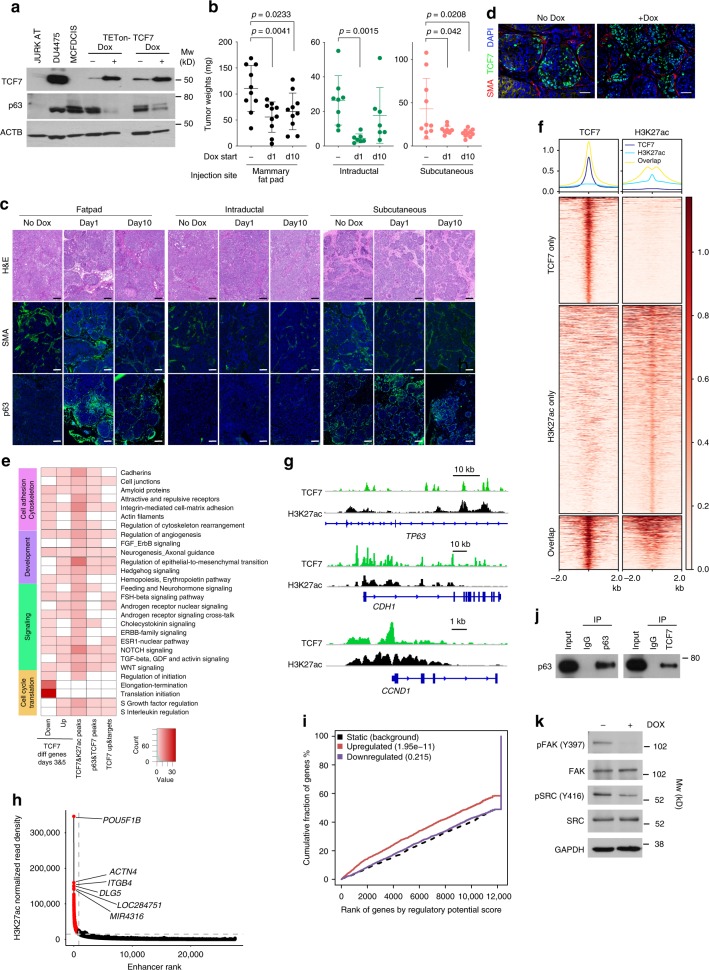
Functional relevance of TCF7 in myoepithelial cells. **a** Immunoblot analysis of p63 and TCF7 expression in parental MCF10DCIS cell line and TCF7 overexpressing TET-inducible derivatives. Jurkat and DU4475 cells were used as positive control for TCF7. ACTB is loading control. **b** Xenograft tumor weight of parental and TCF7 expressing MCF10DCIS cells by mammary fat pad, intraductal or subcutaneous injection. *p*-values indicate statistical significance of difference in tumor weight between groups based on *T*-test. Mean ± SD shown. **c** Hematoxylin–eosin (H&E) staining and immunohistochemical analysis of SMA and p63 expression. Scale bar 100 μm. **d** Immunofluorescence analysis of TCF7 and SMA expression in xenograft tumors of parental and TCF7 expressing MCF10DCIS cells. Scale bar 100 μm. **e** Pathway-enrichment analysis of genes upregulated and downregulated following TCF7 expression in MCF10DCIS cells. Color scale corresponds to −log(*p*-value) of significance of enrichment, calculated by MetaCore enrichment analysis test. **f** Heatmap depicting TCF7 unique and H3K27ac overlapping peaks. The color key is the score of ChIP-seq signal over selected genomic region, the signals across different genomic regions have scaled to the same length. **g** Gene tracks of TCF7 and H3K27ac signals at selected genomic loci. The *x*-axis shows position along the chromosome with gene structures drawn below, whereas *y*-axis shows genomic occupancy in units of rpm/bp. **h** Hockey stick plot depicting changes in super-enhancers in MCF10DCIS cells after TCF7 expression. **i** Integration of differential gene expression and TCF7 targets by BETA analysis. The *p*-value listed in the top left represents the significance of the UP or DOWN group relative to the NON group as determined by the Kolmogorov–Smirnov test. **j** Immunoblot analysis of total cell lysates and TCF7 immunoprecipitates. **k** Immunoblot analysis of MCF10DCIS-TCF7 cells with or without doxycycline (dox) (24 h) for phospho-FAK^Y397^, FAK, phospho-SRC^Y416^, and SRC. GAPDH serves as loading control. Source data are provided as a Source Data file

Although TCF7 and p63 are co-expressed in normal myoepithelial cells, we were not able to detect TCF7 expression in MCF10DCIS cells neither in cell culture nor in xenografts suggesting that MCF10DCIS cells do not fully recapitulate the normal differentiated myoepithelial cell phenotype (Fig. 5a). However, to analyze the effects of p63 and TCF7 co-expression on myoepithelial cell features in the MCF10DCIS model, we expressed HA-tagged TCF7 (long isoform) using a TET-ON expression vector. Induction of TCF7 expression led to a decrease in p63 implying cross-regulation (Fig. 5a). Due to the leakiness of the TET-ON vector we detected TCF7 protein even in unstimulated controls, exhibiting minimal repression of p63 (Fig. 5a), thus, reproducing the TCF7-p63 co-expression observed in normal myoepithelium.

To investigate the effects of TCF7 expression on tumor growth and histology, we performed xenograft assays. Similar to sh*TP63*, we tested mammary fat pad, intraductal, and subcutaneous injections to see how the microenvironment influences tumor growth and histology, and we induced TCF7 at day 1 or day 10 after injection. Expression of TCF7 decreased tumor size regardless of injection site and time of induction, although again, there was a significant difference in tumors size with fat pad tumors being the largest and intraductal the smallest (Fig. 5b). There were significant differences in histology as well, with mixed DCIS-IDC histology in the fat pad, invasive histology in the intraductal group, and DCIS in subcutaneous tumors (Fig. 5c). Interestingly, in the xenografts TCF7 expression was heterogeneous and SMA^+^ myoepithelial cells were largely TCF7 negative (Fig. 5d). In tumors with mixed IDC histology, both p63 and SMA expression were much lower in myoepithelial cells than in more DCIS-resembling areas with nearly complete absence of these cells in intraductal tumors (Fig. 5c). These data suggest that the microenvironment has the most pronounced effect on tumor histology and myoepithelial cell features possibly via regulating p63 and TCF7.

To investigate the genomic targets of TCF7 and the enhancer landscape of MCF10DCIS-TCF7 cells, we analyzed the gene expression profiles of cells at different time points following TCF7 induction and performed ChIP-seq for TCF7 and H3K27ac (Supplementary Data 8). Pathway analysis showed an enrichment of genes upregulated following TCF7 overexpression for cell cycle S phase and G2-M, antigen presentation, proteolysis, DBS repair, mRNA processing, and regulation of angiogenesis, whereas downregulated genes were enriched for translational regulation, cytoskeleton rearrangement, cell adhesion, and FSH-signaling pathways (Fig. 5e). These gene expression changes are consistent with the apparent increase in cell proliferation after TCF7 expression (Supplementary Fig. 4b).

TCF7 ChIP-seq demonstrated 68,592 TCF7-binding sites in MCF10DCIS cells and one-third of these (23,034) overlapped with H3K27ac peaks (Fig. 5f and Supplementary Data 8). Pathway analysis of genes nearest to TCF7 and H3K27ac overlapping peaks demonstrated enrichment in EMT, integrin-mediated cell adhesion, angiogenesis, and NOTCH, WNT, and Hedgehog signaling (Fig. 5e). TCF7 direct targets include *TP63*, *CDH1, CCND1*, and *CCND2* (Fig. 5g). Interestingly, TCF7 is also a direct target of p63, thus, these two TFs cross-regulate each other’s expression.

Analysis of H3K27ac data identified 1,178 SEs in TCF7-HA-expressing cells and most of these were also SEs in MCF10DCIS cells (Fig. 5h and Supplementary Data 6). However, we also identified 135 SEs that were gained after TCF7 expression and many of these were associated with TCF7-binding sites. The TCF7 locus itself was associated with a gained SE, demonstrating that TCF7 self-regulates its own expression, as well as several other TFs (e.g., *FOXO3*, *SMAD2*, *TEAD1*, *IRF2*), and cell cycle regulators (e.g., *CDK6*). The gain of these SEs was coupled with the increased expression of the associated genes, which could contribute to the increase in cell proliferation after TCF7 expression in vitro (Supplementary Fig. 4b). Analysis for CRCs^51^ identified 25 top TFs, mostly overlapping with top CRC-forming TFs in MCF10DCIS cells, but we also observed some differences including gain of *ELF3*, *ETS2*, *IRF2*, and loss of *TP63*. Analysis of interaction networks among these 25 TFs identified a well-connected network with SMAD3, MYC, and SP1 as major hubs (Supplementary Fig. 4c).

To further investigate the effect of TCF7 on transcription, we integrated TCF7 ChIP-seq and RNA-seq data and found that genes upregulated after TCF7 expression were significantly predicted to be TCF7 targets indicating that TCF7 functions as a transcriptional activator in MCF10DCIS cells (Fig. 5i). Pathway analysis of direct TCF7 targets that are upregulated after TCF7 expression showed enrichment in proteolysis, antigen presentation, cell cycle S phase, ESR1 pathway, mitosis, regulation of angiogenesis, and EMT (Fig. 5e).

We also analyzed potential overlap between TCF7 and p63 peaks in MCF10DCIS cells and found that ~25% of p63 peaks overlap with TCF7-binding sites (Supplementary Fig. 4d, e). Most genes associated with these overlapping peaks are stem cell-related, such as WNT, NOTCH, and Hh signaling (Fig. 5e) implying that TCF7 and p63 may cooperate to regulate the proliferation and differentiation of epithelial stem cells. To test if p63 and TCF7 are in the same chromatin complex in MCF10DCIS cells, we performed TCF7 immunoprecipitation followed by p63 immunoblot. We found that a significant fraction of p63 is associated with TCF7, which could explain the significant overlap between p63 and TCF7 peaks (Fig. 5j).

Our xenograft and gene expression data implied that downregulation of *TP63* and overexpression of *TCF7* may have similar functional consequences and that both TFs regulate the expression of ECM and cell adhesion proteins, which in turn regulate p63 protein levels. To determine if the apparent decrease in p63 protein levels after TCF7 overexpression in MCF10DCIS cells could be due to indirect effects through the ECM, we performed immunoblot analysis for FAK and SRC activity. We found that TCF7 expression results in decreased phospho-FAK^Y397^ and phospho-SRC^Y416^ levels (Fig. 5k), which could explain the decrease in p63 protein levels, since inhibition of these pathways had the same effect (Fig. 4f). We also evaluated if decrease in FAK and SRC activity influences cellular migration, invasion, or adhesion to different substrates. We found that while cell migration and invasion were not significantly different between control and sh*TP63* or TCF7-expressing cells, although migration and invasion is very low in MCF10DCIS cells making it difficult to see a decrease, cell adhesion to fibronectin and collagens significantly decreased (Supplementary Fig. 4f, g). These results suggest that p63 and TCF7 cooperate to regulate ECM and cell adhesion, and these reciprocatively regulate the levels and activity of these two TFs (Supplementary Fig. 4h).

## Discussion

*BRCA1* and *BRCA2* germline mutation carriers have an increased risk of breast cancer, but the cellular and molecular basis of this increased risk is still poorly defined. Prior studies described perturbed luminal differentiation and expansion of luminal progenitors in *BRCA1* mutation carriers^33,34,39^. However, the potential impact of *BRCA1/2* germline mutation on myoepithelial cells has not been investigated in detail. In this study, we describe the molecular characterization of CD10^+^ myoepithelial cell population from normal breast tissue of healthy control nulliparous and parous women with no family history of breast cancer, and *BRCA1*/*2* mutation carriers, as well as from DCIS. Based on our integrated analyses of gene expression, enhancer, and p63 and TCF7 genomic target profiles, we determined that normal myoepithelial cell programs are maintained by an interactive TF network orchestrated by p63 and TCF7 in part via their regulation of ECM proteins and cell adhesion.

p63 plays key roles in the formation of epithelia during embryonic development and germline mutations in *TP63* are responsible for multiple syndromes that involve malformations of various epithelial structures, limb deformations, and cleft palate^54^. Some of these individuals also have other abnormalities including hypoplastic mammary glands and/or nipples highlighting the importance of p63 in mammary gland development. Interestingly, several other genes that we identified as co-expressed and/or targets of p63 in myoepithelial cells are also linked to congenital syndromes with skin and facial abnormalities. *IRF6* is co-expressed with p63 in normal CD10^+^CD44^−^ cells and it is the target of both p63 and TCF7 in MCF10DCIS cells. Germline mutations in *IRF6* cause van der Woude syndrome^55^, while its deletion in mice results in skin and limb abnormalities^56^. Germline mutations in *TFAP2A* and *TFAP2B*, TFs also co-expressed with p63 in myoepithelium, cause branchio-oculo-facial and Char syndrome^57^, respectively. Abnormal epithelial cell differentiation, and disorganized cell adhesion and ECM pathways are features of all these syndromes implying that perturbation of the interactive TF network orchestrated by p63 at different points can have similar effects.

TCF7 (a.k.a. TCF1) is a member of the TCF/LEF TF family that are nuclear-binding partners of β-catenin and downstream mediators of WNT signaling^47,58^. APC/WNT/β-catenin signaling is a key regulator of stem/progenitor cell proliferation and survival, and germline mutations of APC predispose to colorectal and, at lower penetrance, breast carcinomas^59^. In animal models, mammary-specific deletion of *Apc* leads to delayed ductal development and metaplastic outgrowths, but these do not progress to neoplasia^60^. However, combined deletion of *Apc* and *Tcf1* (Tcf7) completely abrogates mammary gland development and leads to acanthomas^45^. In our study, we identified TCF7 as a TF co-expressed with p63 in normal myoepithelial cells in control women. Furthermore, p63 and TCF7 also colocalize on the chromatin near genes required for normal myoepithelial cell function including *ACTA2* (smooth muscle actin) and *OXR1* (oxytocin receptor 1). In contrast, in *BRCA1* mutation carriers and in DCIS TCF7 and p63 are expressed in distinct cell types and almost no overlap is detected in their genomic binding. These data suggest that changes in the cellular expression pattern of TCF7 play important roles in breast tumor initiation and progression.

We identified an extensive cross-talk between p63 and TCF7/WNT signaling both in normal myoepithelial cells and also in the MCF10DCIS model, as well as a crosstalk of these pathways with ECM, Hh, and TGFβ signaling (Supplementary Fig. 4h). TCF7 and p63 are direct genomic targets of each other and p63 protein levels decrease with an increase in TCF7 in MCF10DCIS cells due to changes in cell–matrix interaction and pathways regulated by these including FAK and SRC signaling. GLI3, a transcriptional mediator of Hh signaling, is also a direct target of p63, while SMADs, transcriptional mediators of TGFβ signaling are direct targets of TCF7. At the same time, each one of these TFs regulate numerous cell adhesion and ECM proteins, which in turn regulate p63 protein levels. In normal myoepithelial cells p63 and TCF7 are co-expressed in non-carrier women, but the fraction of these cells is decreased in *BRCA1* mutation carriers. Based on the phenotype of the *Tcf1*^*−/−*^ mice demonstrating mammary adenomas in the absence of *Tcf1*, it is possible that the decrease of TCF7 in basal/myoepithelial cells of *BRCA1* mutation carriers may play a role in their higher risk of breast cancer, especially predisposing them to basal/triple-negative tumors. However, functional data obtained in animal models and in the MCF10DCIS model we utilized in this study should be interpreted with caution as neither mice nor the MCF10DCIS model fully recapitulate the expression patterns of p63 and TCF7 observed in normal human breast tissues. Thus, mechanistic studies focusing on myoepithelial cell differentiation would require the development and testing of more faithful models in the future. Mammary organoid cultures^61^ or certain strains of rats that can be manipulated experimentally may potentially be useful in this regard.

DCIS is a precursor of invasive breast cancer, but currently we lack molecular markers to predict the likelihood of progression. We and others have analyzed the gene expression and genetic profiles of DCIS and IDC with the aim of identifying histologic stage-specific markers and drivers of invasive progression^62–65^, but none of the cancer cell-specific markers had shown consistent differences. In contrast, the lack of myoepithelial cells differentiates invasive from in situ disease and DCIS-associated myoepithelial cells show consistent differences compared to normal. Interestingly, many genes differentially expressed between tumor epithelial cells in DCIS and IDC encode cell adhesion and ECM proteins, and the downregulation of one of these (DST) in an intraductal model increased invasive progression^62^ implying that perturbed cell–ECM interactions play an important role in tumor progression. Since normal myoepithelial cells prevent invasive progression by multiple different mechanisms, perturbed myoepithelial cell differentiation, such as changes in TCF7 expression patterns, could potentially be used as longitudinal biomarkers for patient risk stratification.

## Methods

### Cell lines and breast tissue specimens

MCF10ADCIS.com cells were generously provided by Fred Miller (Karmanos Cancer Institute, Detroit, MI, USA) and cultured following the provider’s recommendations. Jurkat (TIB-152) and DU4475 (HTB123) cell lines were purchased from ATCC. Cell line identity was confirmed by short tandem repeats (STR) analysis and the cells were regularly tested for mycoplasma (Venor GeM Mycoplasma Detection Kit, Sigma). Fresh normal and neoplastic breast tissue specimens were collected at Harvard-affiliated hospitals, at John Hopkins University School of Medicine, Baylor-Charles A. Sammons Cancer Center, Hellen Diller Family Comprehensive Cancer Center, Washington University School of Medicine, University of Michigan, Sutter Roseville Medical Center, Seoul National University using protocols approved by the Institutional Review Board at each institution. Tissue samples at Dana-Farber Cancer Institute were collected under Dana-Farber Harvard Cancer Center (DF/HCC) Institutional Review Board (IRB) protocol #93-085 following written informed consent and used in the lab in compliance with DF/HCC IRB protocol #14-400 approved for the use of de-identified tissue samples. The study is compliant with all relevant ethical regulations regarding research involving human participants. Samples were de-identified prior to transport to the laboratory.

### FACS, immunofluorescence, and immunohistochemical analyses

For FACS, single-cell suspensions of human breast epithelial cells were obtained by collagenase IV (1 mg/ml, Worthington, LS004l89bar) hyaluronidase (1 mg/ml, Sigma cat#H3506) digestion in DMEM/F12 followed by trypsinization. Cells were stained with DAPI, and the following antibodies at 1:100 dilution in PBS + 2% BSA solution: CD10-FITC (Fisher Scientific cat#F082601F) or CD10-RPE (DAKO, clone SS2/36, cat#R084801), or CD10 (Biolegend, clone HI10A, cat#312202), CD10-FITC (BD Biosciences; clone HI10a, cat#340925), CD24-Alexa 647 (Biolegend; clone ML5, cat#311110) and CD44-PE (BD Biosciences; clone 515, cat#550989). The analysis was performed on a BD FACS Canto system (BD Biosciences). Gating and subpopulation analysis were performed using FlowJo software.

Immunofluorescence and immunohistochemical analyses were performed following standard protocols for formalin-fixed paraffin-embedded tissues using antibodies CD10 (DAKO, clone 56C6, cat#M0727), TCF7 (Cell Signaling, C63D9, cat#2203S), pan-cytokeratin (DAKO, Clone AE1/AE3, cat#M3515), SMA (Thermo Scientific, Clone 1A4, cat#MS113B), and p63 (Santa Cruz, clone 4A4, cat#sc-8431) at 1:100 dilution in PBS 10% goat serum. Images from multiple areas of each sample were acquired using a Nikon Ti microscope attached to a Yokogawa spinning-disk confocal unit using a 603 plain apo objective, and OrcaER camera controlled by Andor iQ software; or a Leica SP5 confocal scanning microscope or slides were scanned by Servicebio (http://www.servicebio.com). Expression of TCF7 and p63 in metaplastic tumors was scored as follows 0 (negative), 1 (weak), and 2 (strong), whereas the co-expression of the two proteins were scored as 0 (no co-staining), 1 (<50% overlap), and 2 (>50% overlap).

### SAGE-seq, RNA-seq, ChIP-seq sample preparation, and data analysis

A subset of the RNA-seq and ChIP-seq libraries were generated by the Center for Cancer Computational Biology (CCCB), Center for Functional Cancer Epigenetics (CFCE), and Molecular Biology Core (MBC) at Dana-Farber Cancer Institute (DFCI) following manufacturer’s protocols. ChIP for p63, H3K27ac, TCF7, and TCF7-HA was performed using antibodies p63 (abcam, ab735), H3K27ac (abcam, ab4729), TCF7 (Sigma, WH0006932M1), and HA (abcam, ab9110)^66^. For TCF7 and TCF7-HA ChIP-seq, 1 × 10^7^ cells were fixed with 2 mM DSG (Thermo Fisher Scientific cat#20593) for 30 min at room temperature. DSG was then removed and replaced with fixing buffer (50 mM HEPES–NaOH (pH 7.5), 100 mM NaCl, 1 mM EDTA) containing 1% paraformaldehyde (Electron Microscopy Sciences, 15714) and crosslinked for 10 min at 37 °C. For histone modification ChIP-seq, 5 × 10^6^ cells were fixed with 1% paraformaldehyde for 10 min at room temperature. For ER ChIP-seq, 1 × 10^7^ cells were fixed with 1% paraformaldehyde for 10 min at 37 °C. Crosslinking was quenched by adding glycine to a final concentration of 0.125 M. Cells were washed with ice-cold PBS and harvested in PBS. The nuclear fraction was extracted by first resuspending the pellet in 1 ml of lysis buffer (50 mM HEPES–NaOH (pH 8.0), 140 mM NaCl, 1 mM EDTA, 10% glycerol, 0.5% NP-40, and 0.25% Triton X-100) for 10 min at 4 °C. Cells were pelleted, and washed in 1 ml of wash buffer (10 mM Tris–HCl (pH 8.0), 200 mM NaCl, 1 mM EDTA) for 10 min at 4 °C. Cells were then pelleted and resuspended in 1 ml of shearing buffer (10 mM Tris–HCl (pH 8), 1 mM EDTA, 0.1% SDS) and sonicated in a Covaris sonicator. Lysate was centrifuged for 5 min at 14,000 rpm to purify the debris. Then 100 µl of 10% Triton X-100 and 30 µl of 5 M NaCl were added. The sample was then incubated with 20 µl of Dynabeads Protein G (LifeTechnologies,10003D) for 1 h at 4 °C. Primary antibodies were added to each tube and immunoprecipitation (IP) was conducted overnight in the cold room. Cross-linked complexes were precipitated with Dynabeads Protein G for 2 h at 4 °C. The beads were then washed in low salt wash buffer (20 mM Tris–HCl pH 8, 150 mM NaCl, 10 mM EDTA, and 1% SDS) for 5 min at 4 °C, high salt wash buffer (50 mM Tris–HCl pH 8, 10 mM EDTA, and 1% SDS) for 5 min at 4 °C and LiCl wash buffer (50 mM Tris–HCl pH 8, 10 mM EDTA, and 1% SDS) for 5 min at 4 °C. DNA was eluted in elution buffer (100 mM sodium bicarbonate and 1% SDS). Cross-links were reversed overnight at 65 °C. RNA and protein were digested with 0.2 mg/ml RNase A for 30 min at 37 °C followed by 0.2 mg/ml Proteinase K for 1 h at 55 °C. DNA was purified with phenol–chloroform extraction and isopropanol precipitation. ChIP-seq libraries were prepared using the ThruPLEX DNA-seq Kit (Rubicon, cat#R400427) from 1 ng of purified ChIP DNA or input DNA according to the manufacturer’s protocol. ChiLin pipeline 2.0.0^67^ was used for QC and preprocess of the ChIP-seq. We used Burrows–Wheeler Aligner (BWA)^68^ as a read mapping tool, and Model-based Analysis of ChIP-Seq (MACS2)^69^ as a peak caller. Peak annotation was performed using annotatePeaks.pl of the HOMER package v4.9.1 with version hg19 of the human genome^70^. Based on a dynamic Poisson distribution MACS2 can effectively capture local biases in the genome sequence, allowing for more sensitive and robust prediction of binding sites. Unique read for a position for peak calling was used to reduce false-positive peaks, statically significant peaks were finally selected by calculated false discovery rate of reported peaks. The following QC methods were applied to the ChIP-seq data: (1) sequence quality QC, we calculated these scores using the FastQC software (FastQC. http://www.bioinformatics.babraham.ac.uk/projects/fastqc/). A good sequence quality score is ≥ 25; (2) PCR bottleneck coefficient—PBC score ≥ 0.90; (3) percentage overlap with known DHSs derived from the ENCODE project (the minimum required was 70%); (4) peak conservations; (5) number of total peaks (the minimum required was 1000). Deeptools^71^ was used for the heatmap plots. BETA^53^ was used in integrates ChIP-seq of TFs or chromatin regulators with differential gene expression data to infer direct target genes. Super enhancers were called by ROSE^50^ using H3K27ac ChIP-seq data. Core regulator circuits were identified using the superenhancers data using the algorithm developed by the Young lab^51^ and interactions among TFs were visualized using an online tool STRING^52^. Specifically, we identified TFs predicted to form CRCs and selected TFs in the two top scoring CRCs for further analyses. These included 14 TFs in normal myoepithelial cells (ETV5, ETV6, FOXO1, GLI3, MAF, PBX1, RFX2, RUNX1, SMAD3, TCF7L2, TEAD1, TFAP2B, TFAP2C, THRB), 29 TFs in MCF10DCIS cells (RUNX1, SMAD3, RREB1, BHLHE40, TCF7L2, MYC, KLF13, ETV6, SOX9, VDR, ZNF217, GATA3, STAT4, EHF, FOS, TGIF1, FOSL2, RXRA, GLI3, SREBF1, HES1, RARA, TFCP2L1, KLF5, ERF, NFIB, FOS, OSR1, TGIF1), and 25 TFs in MCF10DCIS-TCF7 cells (SMAD3, TEAD1, RREB1, KLF13, BHLHE40, EGR1, MYC, RUNX1, IRF2, TCF7L2, ELF3, RARA, SP1, STAT4, EHF, ETS2, FOS, RXRA, RFX3, FOSL2, FOXO3, TFCP2L1, DLX2, SREBF1, GATA3). These TFs were then loaded into the STRING online tool to assess interactions using default settings: network edges based on evidence, use all active interaction sources (indicated in legend), medium confidence interaction score (0.004), and use only query proteins.

SAGE-seq library construction and sequencing were performed using long iSAGE kit (Invitrogen, cat#T500003) and following the manufacturer’s instructions^39^. SAGE-seq tags were mapped to genes according to the best tag file for long SAGE (available from SAGE genie website at ftp://ftp1.nci.nih.gov/pub/SAGE/HUMAN). Tag counts mapped to the same genes were combined and total counts were normalized to counts per 10 million reads. For human patient samples, SAGE-seq and RNA-seq results were combined, first quantile normalized and then subjected to batch effect removal using the R software comBat package. Cluster analysis was done using the Cluster and TreeView software (available at http://rana.lbl.gov/EisenSoftware.htm) on top 1000 most variedly expressed genes. PCA analysis was performed using the R software pca function and rgl package. Differential gene expression was done using the R software samr and limma packages. The significance of overlap between gene signatures was performed by a hypergeometric test using the R function phyper. For MCF10DCIS cell line raw RNA-seq datasets read alignment, quality control, and data analysis were performed using STAR^72^. Differential expression is called by DEseq2^73^, significant genes were selected based on cutoff of *P*-value < 0.05 and log_2_fold change > 1.

### Cell adhesion, migration, and invasion assays

Cell adhesion assay was performed using CytoSelect^TM^ 48-Well Cell Adhesion Assay (ECM Array, Colorimetric Format) (Cell Biolabs, Inc., cat#CBA-070) according to the manufacturer’s instruction. In brief, MCF10DCIS-TCF7 and MCF10DCIS-sh*TP63* cells were incubated with doxycycline (1 µg/ml) for 24 and 72 h, respectively. 7.5 × 10^4^ cells in serum-free medium supplemented with 0.5% BSA, 2 mM CaCl_2_, 2 mM MgCl_2_, and with or without doxycycline were plated on ECM Adhesion plate and incubated for 1 h in a cell culture incubator. Migration and invasion assays were performed using CytoSelect^TM^ 24-Well Cell Migration and Invasion Assay (8 µm, Colorimetric Format) (Cell Biolabs, Inc., cat#CBA-100-C) according to the manufacturer’s instructions. In brief, MCF10DCIS-TCF7 and MCF10DCIS-sh*TP63* cells were incubated with doxycycline (1 µg/ml) for 24 and 72 h, respectively. 4 × 10^4^ cells in serum-free medium supplemented with 0.5% BSA, 2 mM CaCl_2_, 2 mM MgCl_2_ and with or without doxycycline were plated on invasion or migration insert. Insert was placed on full media. Migration assay was incubated for 24 h and invasion assay for 48 h in a cell culture incubator.

### Immunoblot analyses

Cell lysates were prepared in ice-cold RIPA lysis buffer (50 mM Tris pH 8, 150 mM NaCl, 1% NP40, 0.5% Na-deoxycholate, 0.1% SDS) supplemented with HALTTM Protease and Phosphatase Inhibitor Cocktail (Thermo Fisher Scientific) and sonicated using a Bioruptor^®^ Pico sonication device (diagenode). Protein content was determined with the BCA Protein Assay Kit (Pierce). 20 µg of protein were resolved by SDS–PAGE, transferred to Nitrocellulose membrane (Thermo Fisher Scientific) and blocked in 5% Milk/TBST for 1 h at RT. Primary antibodies were used as follows: anti-p63 (ab735; 1:500; abcam), anti-phospho FAK-Y397 (#8556; 1:500; Cell Signaling Technology), anti-FAK (#3285; 1:1000; Cell Signaling Technology), anti-phospho Src-Y416 (#6943; 1:1000; Cell Signaling Technology), anti-Src (#2109; 1:1000; Cell Signaling Technology), TCF7 (#2203; 1:1000; Cell Signaling Technology), anti-GAPDH (#5174; 1:5000; Cell Signaling Technology), and anti-ACTB (#A2228; 1:1000, Sigma-Aldrich).

### Proliferation assay

1.5 × 10^5^ cells were seeded in 6 cm tissue culture plates in duplicate. Cells were pulsed with BrdU (10 µM) for 1 h next day and stained with FITC-conjugated-anti-BrdU antibody and 7-AAD using the BD BrdU Flow kit (BD, Biosciences, cat#552598) according to the manufacturer’s instructions and analyzed by flow cytometry. Data acquisition was performed on a BD LSR Fortessa Flow Cytometry Analyzer, data analysis was done with Cytobank.

### Mass cytometry (CyTOF)

For CyTOF, antibodies were purchased in carrier-free buffers and conjugated with the respective lanthanide metals by the CyTOF Antibody Resource and Core at Brigham Women’s Hospital, Boston, MA, USA. Single cell suspensions of normal breast tissue from non-carrier and *BRCA1* and *BRCA2* mutation carriers were treated with 50 µM IdU-127 (Fluidigm, cat#201127) for 30 min and 100 µM of the intercalator-103Rh (Fluidigm, cat#201103A) for 15 min at 37 °C in DMEM/F12 medium. Next, 1 × 10^6^ cells of each sample were barcoded using the Cell-ID 20-Plex Pd Barcoding Kit (Fluidigm, cat#201060) according to the manufacturer’s instructions. Barcoded samples were pooled and stained simultaneously. Cells were fixed for 10 min with paraformaldehyde (Electron Microscopy Sciences, cat#50-980-494) at a final concentration of 1.6% followed by Fc-receptor block Human TruStain FcX (Biolegend, cat#422302) for 10 min and surface antibody staining for 30 min at room temperature. Subsequently, cells were permeabilized with methanol for 10 min on ice and incubated with the antibody cocktail for intracellular epitopes for 30 min. Cells were kept at 4 °C overnight in Fix and Perm Buffer (Fluidigm, cat#201067) supplemented with Intercalator-IR (Fluidigm, cat#201192A) 1:2000. Prior to analysis cells were washed with water, resuspended in water containing EQ™ Four Element Calibration Beads (Fluidigm, cat#201078) (1:10) and filtered through a 35 µm strainer. Samples were acquired at a CyTOF Helios instrument (Fluidigm), normalized as previously described^74^ and analyzed with Cytobank (Cytobank, Inc., Mountain View, CA). For all washes during staining Cell Staining Media (PBS with 0.5% BSA, 0.02% NaN3) was used. For analysis all files for normal (*n* = 6), *BRCA1* (*n* = 6), and *BRCA2* *(n* = 7) samples have been concatenated to create a single normal, *BRCA1* and *BRCA2* file, respectively. Files have been gated for singlets, viable (defined as Rh-103 negative) and non-apoptotic (cleaved PARP negative). To exclude contaminating immune cells (defined as CD45^+^), files have further been gated for CD45^−^ population. viSNE analysis has been performed using this population or the myoepithelial population defined as CK8/18 negative, E-Cadherin^+^ and SMA^+^. For all viSNE analysis the following markers were used for clustering: PR, CD10, CD44, MUC1, CD24, Vimentin, Epcam, CK8/18, SMA, GATA-3, ER, AR, HER2, CK5, E-Cadherin, CD49f. Antibodies used for CYTOF (metal): Rabbit monoclonal anti-PR a/b (141Pr), Cell Signaling Technology Cat#8757; Mouse monoclonal anti-CD10 (142Nd) BD Biosciences Cat#555373; Rat monoclonal anti-CD44 (143Nd) Biolegend Cat#103002; Mouse monoclonal anti-cyclin D3 (144Nd) Abcam Cat#ab28283; Mouse monoclonal anti-MUC1 (145Nd) Biolegend Cat#355602; Mouse monoclonal anti-LAMP2 (146Nd) Biolegend Cat#354302; Mouse monoclonal anti-CDK4 (147Sm) BD Biosciences Cat#559677; Rabbit monoclonal anti-PTEN (148Nd) Cell Signaling Technology Cat#9559; Rabbit monoclonal anti-E-Cadherin (149Sm) Cell Signaling Technology Cat#3195; Mouse monoclonal anti-EpCAM (150Nd) Biolegend Cat#324202; Mouse monoclonal anti-HER2 (151Eu) BD Biosciences Cat#554299; Rabbit polyclonal anti-CK5 (152Sm) Abcam Cat#ab53121; Mouse monoclonal anti-CD24 (153Eu) Biolegend Cat#311102; Mouse monoclonal anti-CDK1 (154Sm) Biolegend Cat#626901; Rabbit monoclonal anti-CDK6 (155Gd)

Cell Signaling TechnologyCat#13331; Rabbit monoclonal anti-p63 (158Gd) Abcam Cat#ab124762; Rabbit monoclonal anti-TCF7 (159Tb) Cell Signaling Technology Cat#2203; Rabbit monoclonal anti-AR (160Gd) Cell Signaling Technology Cat#5153; Mouse monoclonal anti-Cyclin A (161Dy) BD Biosciences Cat#554175; Mouse monoclonal anti-Ki-67 (162Dy) BD Biosciences Cat#550609; Mouse monoclonal anti-SMA (163Dy) Thermo Fisher Scientific Cat#14-9760-82; Mouse monoclonal anti-cPARP (164Dy) BD Biosciences Cat#552596; Rabbit monoclonal anti-Vimentin (165Ho) Cell Signaling Technology Cat#5741; Rat monoclonal anti-GATA3 (166Er) eBioscience Cat#14-9966-80; Rabbit monoclonal anti-p21 (167Er) Cell Signaling Technology Cat#2947; Rabbit monoclonal anti-phospho-AKT Ser473 (168Er) Cell Signaling Technology Cat#4060; Rabbit monoclonal anti-phospho-STAT3 Tyr705 (169Tm) Cell Signaling Technology Cat#9145; Rabbit monoclonal anti-EGFR (170Er) Cell Signaling Technology Cat#4267; Rabbit monoclonal anti-phospho-SMAD2 Ser465/467/Smad3 Ser423/425 (171Yb) Cell Signaling Technology Cat#8828; Rabbit monoclonal anti-ERα (172Yb) Cell Signaling Technology Cat#13258; Rat monoclonal anti-CD49f (173Yb) Biolegend Cat#313602; Rabbit monoclonal anti-phospho-STAT5 Tyr694 (174Yb) Cell Signaling Technology Cat#4322; Rabbit monoclonal anti-phospho-S6 Ser235/236 (175Lu) Cell Signaling Technology Cat#4858; Mouse monoclonal anti-CK8/18 (176Yb) Cell Signaling Technology Cat#4546; Mouse monoclonal anti-CD45 (156Gd) BD Biosciences Cat#555480.

### shRNA plasmids and lentivirus production

pLKO shRNA vectors for control GFP (clone 437) and *TP63* (clones 6502, 6504, and 6506) were obtained from the Broad Institute RNAi consortium (TRC). To express doxycycline-inducible shRNAs, annealed oligos (LacZ and TP63) were cloned into pLKO-tet-on lentiviral vector (kindly provided by Dr. Alex Toker, Beth-Israel Deaconess Medical Center, Boston, MA). For expression of TCF7, HA tagged full-length TCF7 cDNA was obtained from pcDNA-HA-TCF1 plasmid purchased from Addgene^75^. cDNA was inserted into pLenti6.3 Gateway compatible lentiviral vector (Life Technologies, cat#V53306). To produce lentiviral supernatants, HEK293T cells were co-transfected with shRNA or expression vectors, VSVG, and pDG8.91 using Fugene 6 (Roche, cat#11988387001). The targeting cells were infected with the viral supernatant containing 8 µg/ml polybrene. 48 h post-infection, the target cells were exposed to puromycin (2 µg/ml) to select for infected cells.

### Mouse xenograft assays

Female NSG mice (8 weeks old) were purchased from The Jackson Laboratory and maintained in pathogen-free conditions. Tumors were induced by mammary fat pad, intraductal and subcutaneous bi-lateral injections of MCF10DCIS-sh*TP63* (45 mice, 5 mice per treatment group) or MCF10DCIS-TCF7 (45 mice, 5 mice per treatment group) cells (2 × 10e5 in 50 µL, 1 × 10e5 in 20 µL, and 2 × 10e5 in 100 µL, respectively) resuspended in DMEM-F12 medium/Matrigel Growth Factor Reduced Basement Membrane Matrix, Phenol Red-Free (Fisher Scientific, cat#CB356238) in a 1:1 ratio. Treatment groups received doxycycline rodent chow (2000 ppm). Animal experiments were performed by the Lurie Family Imaging Center following protocols approved by the Dana–Farber Cancer Institute Animal Care and Use Committee. The study is compliant with all relevant ethical regulations regarding animal research. Mice were euthanized and tumors collected 21 days after injection.

### Statistical analyses

Statistical significance of differences in tumor weight and cell adhesion were determined by based on *T*-test. For assessing the association among cell subtype (Figs. 1h and 2f: p63^−^TCF7^-^, p63^+^TCF7^+^, TCF7^+^p63^−^, p63^+^TCF7^−^; Fig. 2b: CD10^+^CD44^−^, CD10^+^CD44^+^) and condition Fig. 1i: Control, *BRCA1*, *BRCA2*; Fig. 2b: Normal and DCIS; Fig. 2f: DCIS-HG, DCIS-LG, DCIS-IDC), we first conducted Pearson’s chi-squared tests among averages of estimated cell counts across replicates. For computing the *P*-values in the main figures, we used a conservative estimate of 100 cells. With increasing population size, the power of identifying associations also increases: for 150 cells, *p*-values of 1.485e−07 (Fig. 1h) and 8.998e−06 (Fig. 2f); for 200 cells: 1.864e−10 (Fig. 1h) and 8.713e−08 (Fig. 2f). For Fig. 1h and population size of 100, we simulated the *p*-value, since the count for Control p63-TCF7- was only 2. Next, in order to take into account the variation among replicates (which is not considered by the chi-squared test), we employed methods specific to Compositional Data Analysis^76^. These methods are needed because of the negative correlations artificially introduced by the structure of these data: since the amount of every cell subtype is limited to the whole, if the percentage of one subtype of cells increases, the amounts of other subtypes must decrease. Therefore, we first evaluated whether the cell subtype composition can be accurately predicted given the condition, by using Dirichlet regression^77^. The Dirichlet distribution is a multivariate generalization of the beta distribution and is commonly employed for modeling compositional data^78^. However, likely because of the low number of replicates, as well as the large variation among replicates, in all cases (data from Figs. 1h and 2b, f) the effect sizes were small, and the large majority of predictors were not significant. We further evaluated whether the condition can be accurately predicted given the cell subtype composition, by using Multinomial logistic regression. Prior to this, the cell subtype compositions were transformed to a *D*−1 dimensional space (where *D* is the dimension of the original data), by using the isometric log transform^79^, commonly applied to compositional data. Likely from the same reasons described above, in almost all cases the predictors were not significant, with the sole exception of the ratio of CD10^+^CD44^+^ cells in separating DCIS from Normal, for which the effect size was 1.01, and the *p*-value 0.063.

### Reporting summary

Further information on research design is available in the Nature Research Reporting Summary linked to this article.

## Supplementary information

Supplementary Information

Description of Additional Supplementary Files

Supplementary Data 1

Supplementary Data 2

Supplementary Data 3

Supplementary Data 4

Supplementary Data 5

Supplementary Data 6

Supplementary Data 7

Supplementary Data 8

Reporting Summary

Source Data

## Data Availability

All raw genomic data was deposited to GEO under accession number GSE113909. The source data underlying Figs. 1b, h, 2b, f, h, 4b, 5b and Supplementary Figs. 4b, f, g and the full-size images of immunoblots depicted in Figs. 4d, e, f, 5a, j, k and Supplementary Fig. 3a are included in the Source Data file. Detailed protocols and reagents described in this manuscript are available from the corresponding author upon request.
