# Supplementary material for: Perturbed myoepithelial cell differentiation in BRCA mutation carriers and in ductal carcinoma in situ
Source: Nat Commun. 2019 Sep 13;10:4182. doi: 10.1038/s41467-019-12125-5 (PMC6744561; doi:10.1038/s41467-019-12125-5)
Supplement: Supplementary file 1 — Supplementary Information [file 41467_2019_12125_MOESM1_ESM.pdf]

**Supplementary Information**

# Perturbed myoepithelial cell differentiation in *BRCA* mutation carriers and in ductal carcinoma *in situ* (DCIS)

Ding et al.

## SUPPLEMENTARY FIGURES

**Supplementary Figure 1. Heterogeneity of CD10$^+$ cell populations in normal breast tissues. a**, Schematic outline of the purification procedure and phenotype of the cells in cell culture. **b,** Combined analysis of cells for the expression of CD10 and CD44 cell surface markers and ALDH enzymatic activity. Left panel indicates staining of cells for CD10 and CD44 whereas right panel shows ALDH activity of cells selected based on difference in CD10 and CD44 expression. ALDH activity was assessed with and without DAEB (ALDH inhibitor). **c**, Gating strategy used for the quantification of CD10$^+$CD44$^+$ and CD10$^+$CD44$^-$ cells. Single cell suspension derived from organoids were stained with viability dye-pacific blue, CD24-APC, CD44-PE, and CD10-FITC. Each single-color control was run in parallel to ensure proper compensation. Cells were gated using forward scatter (FSC) and side scatter (SSC) and then gated for the pacific blue negative viable cells. Next, CD24-APC$^+$ cells were gated and sorted for other studies. The remaining CD24-APC negative cells were gated for CD10-FITC$^+$CD44-PE$^+$ and CD10-FITC$^+$CD44-PE$^-$ cells and sorted. **d**, viSNE maps of CyTOF analysis of normal breast tissues (CD45 negative population) colored for the indicated markers. Color scale indicates minimum and maximum values of expression in both panels (**d** and **e**). **e**, viSNE maps of CyTOF analysis of myoepithelium (CK8/18 negative, E-Cadherin$^+$, SMA$^+$ population) colored for the indicated markers. **f**, Heatmap depicting unsupervised clustering of samples based on the expression of top 1,000 most variable genes. Scale indicates relative log2 fold changes of gene expression, white is median of all samples and red/blue are relative to median of all samples. **g**, Pathways enriched in genes differentially expressed between the indicated groups of samples. Color scale corresponds to -log (p-value) of significance of enrichment, calculated by MetaCore Enrichment Analysis test. **h**, Gene tracks (189.501-189.533Mb) depicting *TP63* isoforms in normal breast and in MCF10DCIS cells. **i**, Gene tracks (133.448-133.486Mb) depicting *TCF7* isoforms in normal breast and in MCF10DCIS cells.

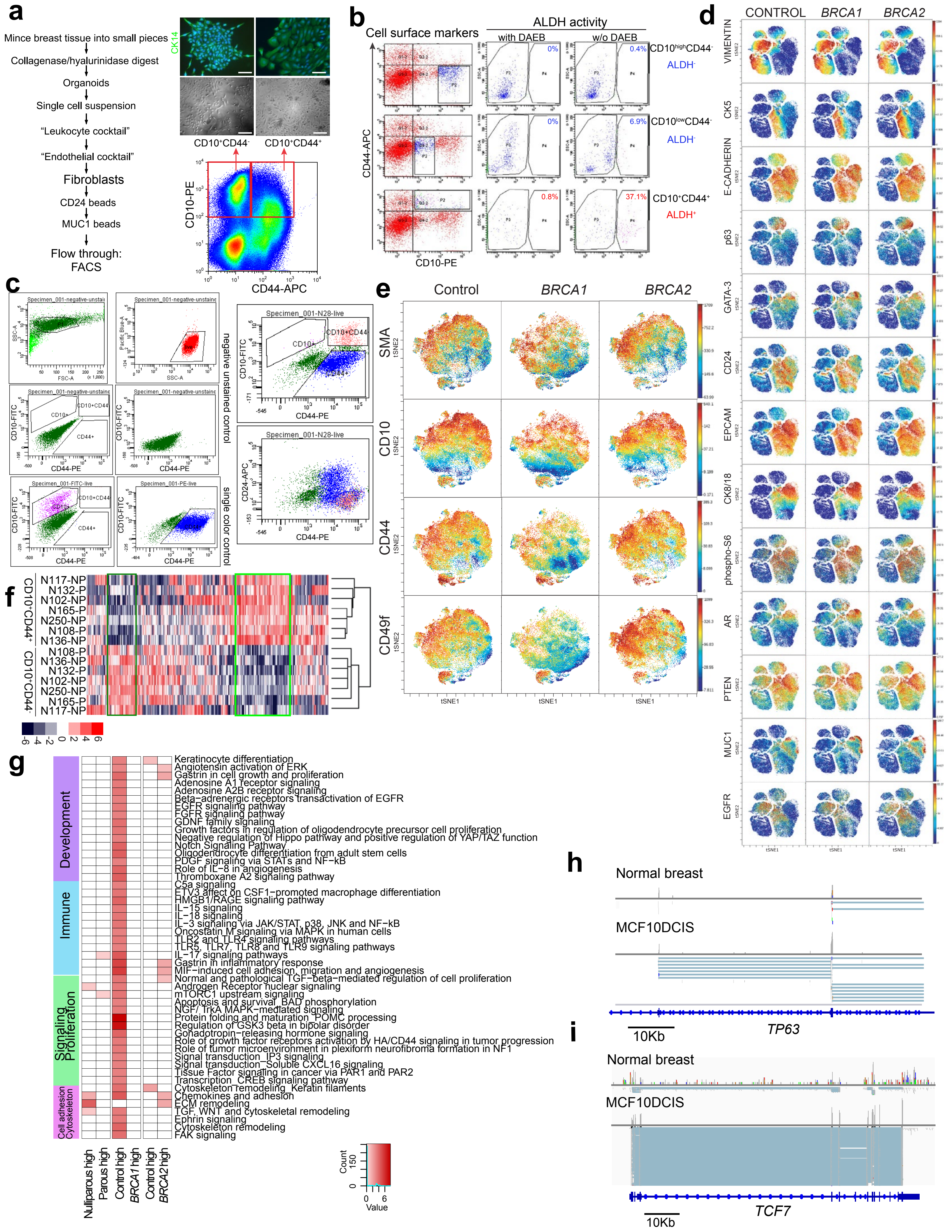
a
Mince breast tissue into small pieces
Collagenase/hyalurinidase digest
Organoids
Single cell suspension
"Leukocyte cocktail"
"Endothelial cocktail"
Fibroblasts
CD24 beads
MUC1 beads
Flow through:
FACS
CK14
CD10+CD44-
CD10+CD44+
CD10-PE
CD44-APC
b
Cell surface markers
ALDH activity
with DAEB
w/o DAEB
CD44-APC
CD10-PE
0%
0.4%
CD10highCD44-
ALDH-
0%
6.9%
CD10lowCD44-
ALDH-
0.8%
37.1%
CD10+CD44+
ALDH+
c
Specimen_001-negative-unstained
Specimen_001-N28-live
Specimen_001-FITC-live
Specimen_001-PE-live
FSC-A
SSC-A
Pacific Blue-A
CD10-FITC
CD44-PE
CD24-APC
CD10+
CD10+CD44+
CD44+
negative unstained control
single color control
d
CONTROL
BRCA1
BRCA2
VIMENTIN
CK5
E-CADHERIN
p63
GATA-3
CD24
EPCAM
CK8/18
phospho-S6
AR
PTEN
MUC1
EGFR
tSNE1
tSNE2
e
Control
BRCA1
BRCA2
SMA
CD10
CD44
CD49f
tSNE1
tSNE2
f
CD10+CD44+
CD10+CD44-
N117-NP
N132-P
N102-NP
N165-P
N250-NP
N108-P
N136-NP
N108-P
N136-NP
N132-P
N102-NP
N250-NP
N165-P
N117-NP
-6 -4 -2 0 2 4 6
g
Development
Immune
Signaling Proliferation
Cell adhesion Cytoskeleton
Keratinocyte differentiation
Angiotensin activation of ERK
Gastrin in cell growth and proliferation
Adenosine A1 receptor signaling
Adenosine A2B receptor signaling
Beta-adrenergic receptors transactivation of EGFR
EGFR signaling pathway
FGFR signaling pathway
GDNF family signaling
Growth factors in regulation of oligodendrocyte precursor cell proliferation
Negative regulation of Hippo pathway and positive regulation of YAP/TAZ function
Notch Signaling Pathway
Oligodendrocyte differentiation from adult stem cells
PDGF signaling via STATs and NF-kB
Role of IL-8 in angiogenesis
Thromboxane A2 signaling pathway
C5a signaling
ETV3 affect on CSF1-promoted macrophage differentiation
HMGB1/RAGE signaling pathway
IL-15 signaling
IL-18 signaling
IL-3 signaling via JAK/STAT, p38, JNK and NF-kB
Oncostatin M signaling via MAPK in human cells
TLR2 and TLR4 signaling pathways
TLR5, TLR7, TLR8 and TLR9 signaling pathways
IL-17 signaling pathways
Gastrin in inflammatory response
MIF-induced cell adhesion, migration and angiogenesis
Normal and pathological TGF-beta-mediated regulation of cell proliferation
Androgen Receptor nuclear signaling
mTORC1 upstream signaling
Apoptosis and survival_BAD phosphorylation
NGF/ TrkA MAPK-mediated signaling
Protein folding and maturation_POMC processing
Regulation of GSK3 beta in bipolar disorder
Gonadotropin-releasing hormone signaling
Role of growth factor receptors activation by HA/CD44 signaling in tumor progression
Role of tumor microenvironment in plexiform neurofibroma formation in NF1
Signal transduction_IP3 signaling
Signal transduction_Soluble CXCL16 signaling
Tissue Factor signaling in cancer via PAR1 and PAR2
Transcription_CREB signaling pathway
Cytoskeleton remodeling_Keratin filaments
Chemokines and adhesion
ECM remodeling
TGF, WNT and cytoskeletal remodeling
Ephrin signaling
Cytoskeleton remodeling
FAK signaling
Nulliparous high
Parous high
Control high
BRCA1 high
Control high
BRCA2 high
Count
Value
h
Normal breast
MCF10DCIS
10Kb
TP63
i
Normal breast
MCF10DCIS
TCF7
10Kb

**Supplementary Figure 2. Gene expression and p63 and TCF7 binding patterns in DCIS and *BRCA* mutation carriers. a**, Heatmap depicting unsupervised clustering of normal and DCIS-associated CD10+ cells based on the expression of top 1,000 most variable genes. Scale indicates relative log2 fold changes of gene expression, white is median of all samples and red/blue are relative to median of all samples. **b**, Venn diagrams indicating the numbers of p63 and TCF7 binding sites which are unique in control, and in *BRCA1* or *BRCA2* mutation carriers, or overlap between groups.

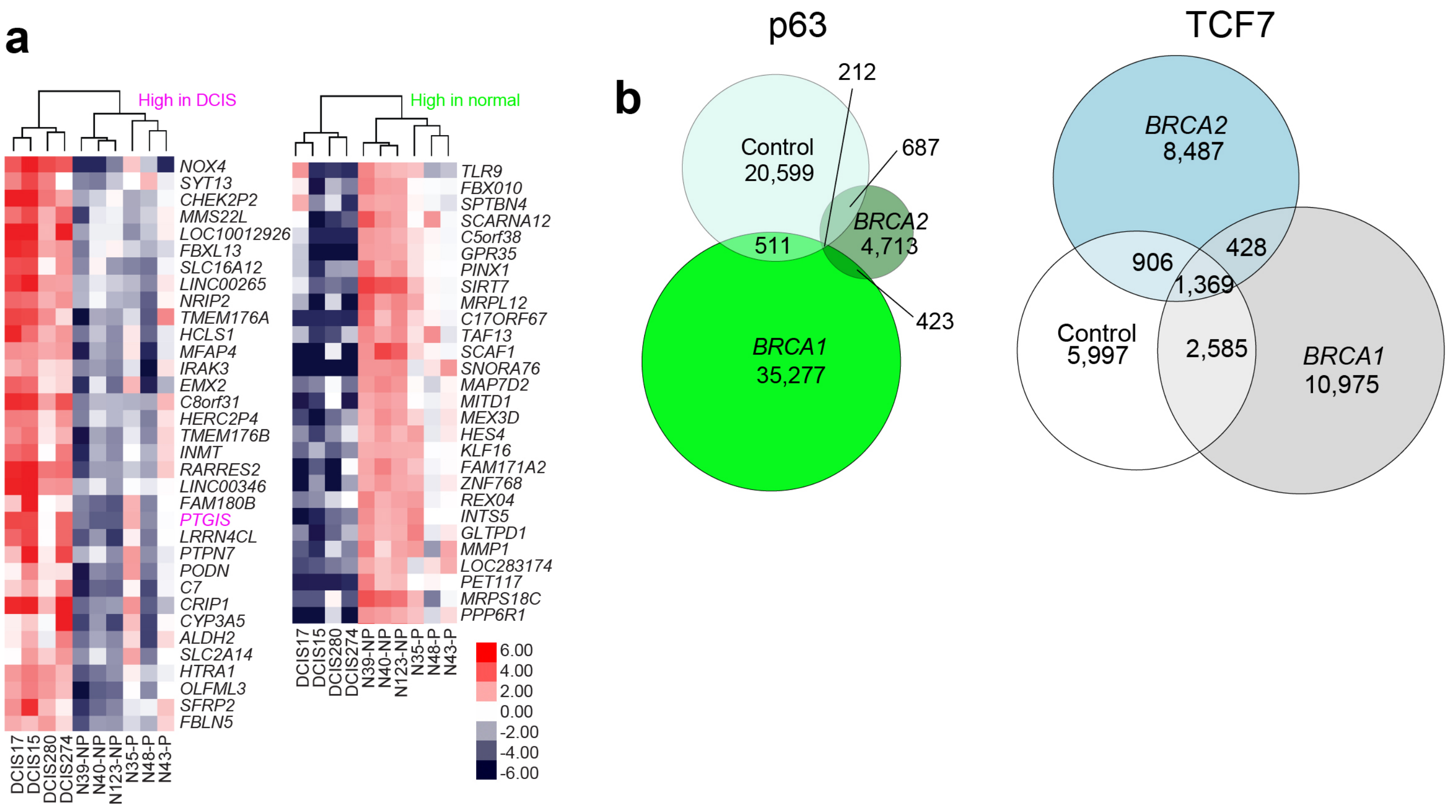

**Supplementary Figure 3. Genomic targets of p63 in MCF10DCIS cells. a,** Immunoblot analysis of p63 and TCF7 expression in MCF10DCIS cells and three different sh*TP63*-expressing derivatives. Jurkat cells were used as positive control for TCF7. ACTB is loading control. **b**, Pathways enriched in genes associated with p63 and H3K27ac overlapping peaks. **c**, Associations between SE ranking, p63 binding sites, and gene expression levels. **d**, Pathway enrichment analysis of p63 genomic targets downregulated in MCF10DCIS cells after induction of sh*TP63* by Dox.

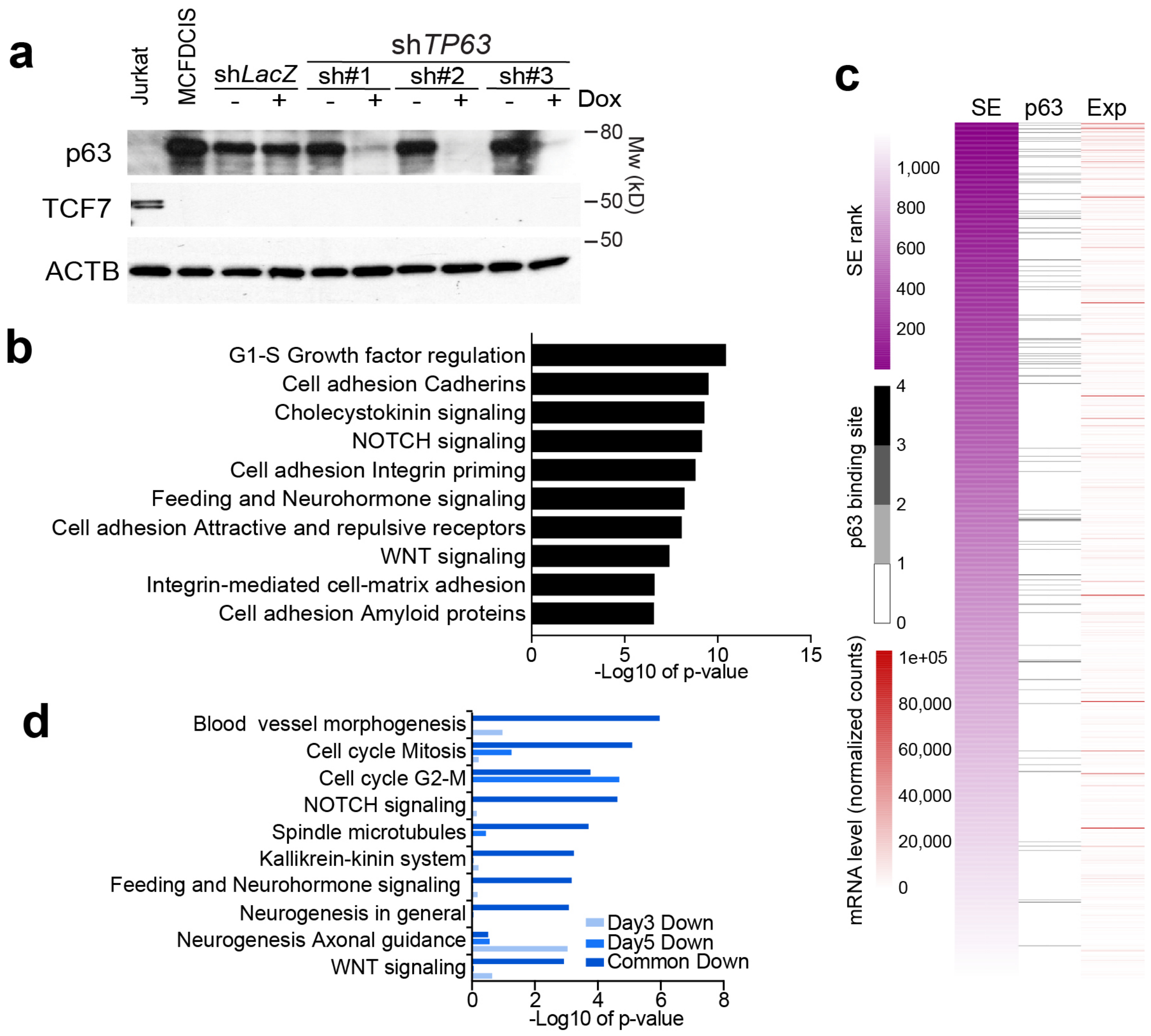

**Supplementary Figure 4. Genomic targets of TCF7 in MCF10DCIS cells. a**, Gating strategy for the identification of cell cycle distribution. Cells were first gated based on 7-AAD-height/7-AAD-area to identify single cells (gate: singlets). Following, single cells were shown for BrdU-FITC and 7-AAD-A and gated as S-Phase (BrdU-FITC-positive), G1-phase (BrdU-FITC-negative; single 7-AAD content) and G2/M-phase (BrdU-FITC-negative; double 7-AAD content). **b**, Relative fraction of cells in different phases of the cell cycle before and after 5 days of doxycycline treatment to induce sh*TP63* and *TCF7*, respectively. **c**, Predicted protein interaction network of TFs identified as core transcriptional regulatory circuits in MCF10DCIS cells expressing TCF7. Legend indicates the source of data used to determine interactions. TFs that are not part of the network were removed. **d**, Heatmap depicting p63 and TCF7 unique peaks and overlapping peaks in MCF10DCIS cells. **e**, Venn diagram indicating the numbers of p63 and TCF7 unique and overlapping binding sites in MCF10DCIS cells. **f**, Dot plot depicting results of cell migration and invasion experiments at different time points following the induction of shTP63 or TCF7 by doxycycline (DOX). **g**, Dot plot depicting results of cell adhesion to different matrices experiments at different time points following the induction of shTP63 or TCF7 by doxycycline (DOX). P-values indicate statistical significance of difference to no DOX determined by t-test. **h**, Simplified model of pathway interactions. Source data are provided as a Source Data file.

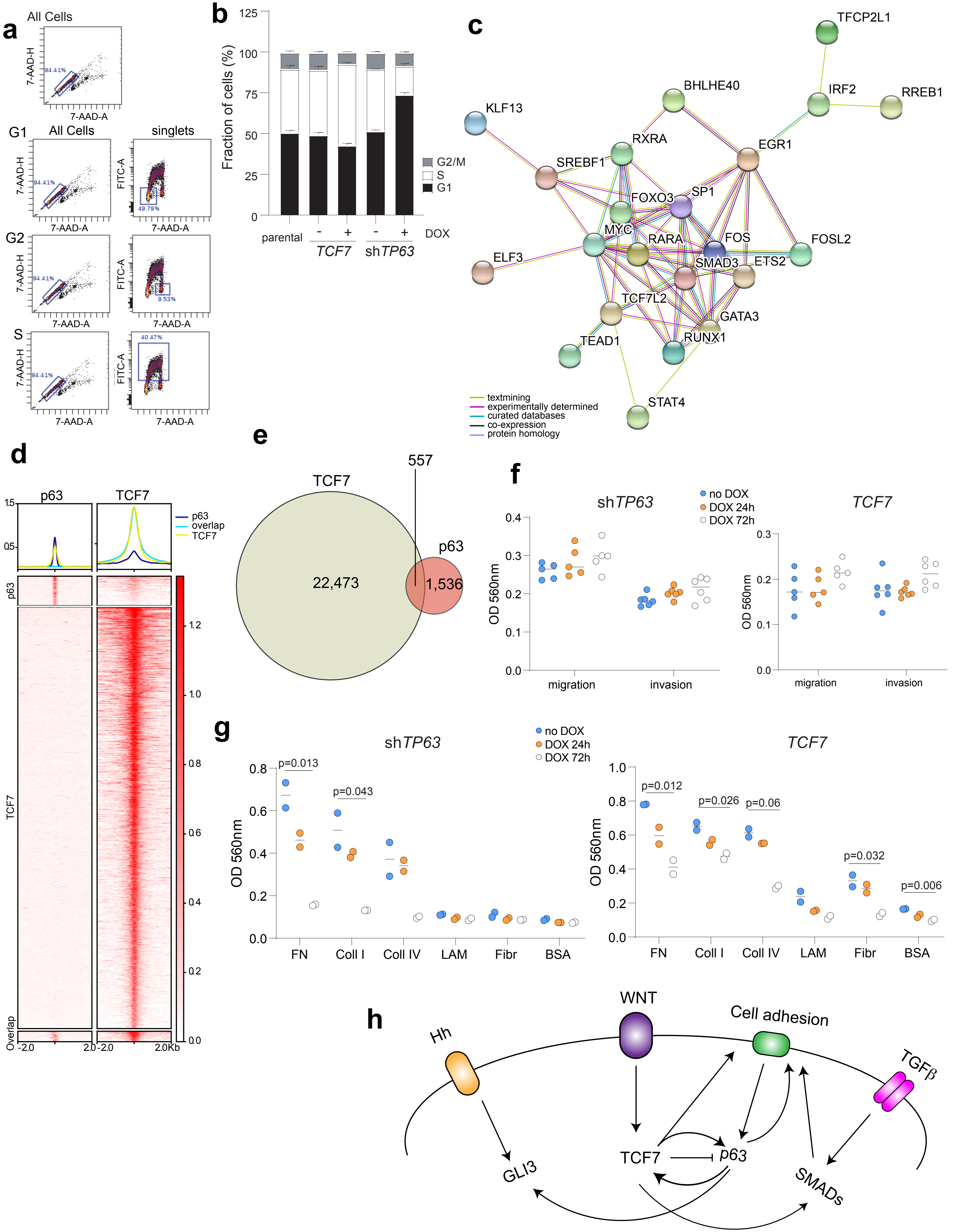
a
All Cells
7-AAD-H
7-AAD-A
94.41%
G1
All Cells
singlets
FITC-A
49.79%
G2
9.53%
S
40.47%
b
Fraction of cells (%)
125
100
75
50
25
0
G2/M
S
G1
parental
-
+
DOX
TCF7
shTP63
c
TFCP2L1
BHLHE40
IRF2
RREB1
KLF13
RXRA
EGR1
SREBF1
FOXO3
SP1
MYC
RARA
FOS
FOSL2
ELF3
SMAD3
ETS2
TCF7L2
GATA3
TEAD1
RUNX1
STAT4
textmining
experimentally determined
curated databases
co-expression
protein homology
d
p63
TCF7
p63
overlap
TCF7
1.5
0.5
1.2
1.0
0.8
0.6
0.4
0.2
0.0
Overlap
-2.0
2.0
2.0Kb
e
557
TCF7
p63
22,473
1,536
f
shTP63
TCF7
no DOX
DOX 24h
DOX 72h
OD 560nm
0.4
0.3
0.2
0.1
0.0
migration
invasion
g
shTP63
TCF7
no DOX
DOX 24h
DOX 72h
OD 560nm
p=0.013
p=0.043
p=0.012
p=0.026
p=0.06
p=0.032
p=0.006
0.8
0.6
0.4
0.2
0.0
1.0
FN
Coll I
Coll IV
LAM
Fibr
BSA
h
WNT
Cell adhesion
Hh
TGFβ
GLI3
TCF7
p63
SMADs
