# Supplementary material for: Perturbed myoepithelial cell differentiation in BRCA mutation carriers and in ductal carcinoma in situ
Source: Nat Commun. 2019 Sep 13;10:4182. doi: 10.1038/s41467-019-12125-5 (PMC6744561; doi:10.1038/s41467-019-12125-5)
Supplement: Supplementary file 2 — Description of Additional Supplementary Files [file 41467_2019_12125_MOESM2_ESM.pdf]

Description of Additional Supplementary Files

File Name: Supplementary Data 1
Description: **Tissue samples used for the study.** The excel file incudes: the information of each human and cell line sample that was used in this study, including sample ID, cell type, tissue type, histology, mutation, age, parity status, and specific experiment for which it was used.

File Name: Supplementary Data 2
Description: **Genes differentially expressed between $CD10^{+}CD44^{+}$ and $CD10^{+}CD44^{-}$ cells.** The excel file includes the following worksheets: the common differentially expressed genes in nulliparous and parous breast cells (common), genes differentially expressed only in nulliparous (nulliparous) or parous (parous) samples, and differentially expressed transcription factors. Gene symbol and description, expression value in each sample, pseudo and average fold change, p-value of difference, and if it is a genomic target of p63 or TCF7 (target or non-target) are listed for each gene.

File Name: Supplementary Data 3
Description: **Genes differentially expressed in $CD10^{+}$ cells from normal breast of control non-carrier women and *BRCA1* or *BRCA2* mutation carriers.** The excel file incudes: genes differentially expressed in $CD10^{+}$ cells from control non-carrier and *BRCA1* or *BRCA2* mutation carrier samples. Gene symbol and description, expression value in each sample, pseudo fold change, p-value of difference, and if it is a genomic target of p63 or TCF7 (target or non-target) are listed for each gene.

File Name: Supplementary Data 4
Description: **Genes differentially expressed in $CD10^{+}$ cells from normal breast and DCIS.** Gene symbol and ID, expression value in each sample, and fold change.

File Name: Supplementary Data 5
Description: **Genomic targets of p63 and TCF7 in normal myoepithelial cells.** The excel file incudes: peak ID, chromosome location, loci, annotation of the nearest genes. The targets are listed for each sample in a separate worksheet.

File Name: Supplementary Data 6
Description: **Super-enhancers (SEs) in normal myoepithelial and MCF10DCIS cells.** The excel file incudes: genomic location of each SE in the different cell types, including peak ID, chromosome location, loci, constituent size, enhancer rank, and super enhancer mapped to the closest genes.

File Name: Supplementary Data 7
Description: **Genes differentially expressed 3 or 5 days after induction of p63 knockdown in MCF10DCIS cells.** The excel file incudes: significantly differentially expressed genes with log2 fold change and padj value, indication of whether the differentially expressed genes are p63 targets or not, and association with H3K27ac peaks.

File Name: Supplementary Data 8
Description: **Genes differentially expressed 3 or 5 days after induction of TCF7 overexpression in MCF10DCIS cells.** The excel file incudes: significantly differentially expressed genes with log2 fold change and padj value, indication of whether the differentially expressed genes are TCF7 targets or not, and association with H3K27ac peaks.
