# Supplementary material for: Perturbed myoepithelial cell differentiation in BRCA mutation carriers and in ductal carcinoma in situ
Source: Nat Commun. 2019 Sep 13;10:4182. doi: 10.1038/s41467-019-12125-5 (PMC6744561; doi:10.1038/s41467-019-12125-5)
Supplement: Supplementary file 11 — Reporting Summary [file 41467_2019_12125_MOESM11_ESM.pdf]

nature research

Corresponding author(s): Kornelia Polyak

Last updated by author(s): Jul 31, 2019

Reporting Summary

Nature Research wishes to improve the reproducibility of the work that we publish. This form provides structure for consistency and transparency in reporting. For further information on Nature Research policies, see Authors & Referees and the Editorial Policy Checklist.

Statistics

For all statistical analyses, confirm that the following items are present in the figure legend, table legend, main text, or Methods section.

n/a	Confirmed	
☐	☒	The exact sample size (*n*) for each experimental group/condition, given as a discrete number and unit of measurement
☐	☒	A statement on whether measurements were taken from distinct samples or whether the same sample was measured repeatedly
☐	☒	The statistical test(s) used AND whether they are one- or two-sided<br>*Only common tests should be described solely by name; describe more complex techniques in the Methods section.*
☐	☒	A description of all covariates tested
☐	☒	A description of any assumptions or corrections, such as tests of normality and adjustment for multiple comparisons
☐	☒	A full description of the statistical parameters including central tendency (e.g. means) or other basic estimates (e.g. regression coefficient) AND variation (e.g. standard deviation) or associated estimates of uncertainty (e.g. confidence intervals)
☐	☒	For null hypothesis testing, the test statistic (e.g. *F*, *t*, *r*) with confidence intervals, effect sizes, degrees of freedom and *P* value noted<br>*Give P values as exact values whenever suitable.*
☒	☐	For Bayesian analysis, information on the choice of priors and Markov chain Monte Carlo settings
☒	☐	For hierarchical and complex designs, identification of the appropriate level for tests and full reporting of outcomes
☐	☒	Estimates of effect sizes (e.g. Cohen's *d*, Pearson's *r*), indicating how they were calculated

Our web collection on statistics for biologists contains articles on many of the points above.

Software and code

Policy information about availability of computer code

Data collection	Image analysis was performed on single or montage images captured by confocal microscopy (Yokogawa spinning disk confocal/TIRF system, for human tissue samples) or performed by Service Bio (xenograft samples). CyTOF data were acquired at a CyTOF Helios instrument (Fluidigm). FACS data were acquired M Fortessa and M Aria II SORP UV. ChIP-seq and RNA-seq were performed using Illumina Nextseq.
Data analysis	Statistical analyses were performed using GraphPad Prism software or R. SAGEseq data was analyzed using SAGE Genie, RNAseq experiments were analyzed using STAR and DeSeq2 R package and ChIPseq data was analyzed using ChiLin pipeline 2.0.0, MACS2, Deeptools and ROSE.

For manuscripts utilizing custom algorithms or software that are central to the research but not yet described in published literature, software must be made available to editors/reviewers. We strongly encourage code deposition in a community repository (e.g. GitHub). See the Nature Research guidelines for submitting code & software for further information.

Data

Policy information about availability of data

All manuscripts must include a data availability statement. This statement should provide the following information, where applicable:

- Accession codes, unique identifiers, or web links for publicly available datasets
- A list of figures that have associated raw data
- A description of any restrictions on data availability

All raw genomic data was deposited to GEO under accession number GSE113909. Secure token for reviewer access while it remains in private status: cfyxmkkoxrmtrux

Field-specific reporting

Please select the one below that is the best fit for your research. If you are not sure, read the appropriate sections before making your selection.

☒ Life sciences ☐ Behavioural & social sciences ☐ Ecological, evolutionary & environmental sciences

For a reference copy of the document with all sections, see nature.com/documents/nr-reporting-summary-flat.pdf

Life sciences study design

All studies must disclose on these points even when the disclosure is negative.

Sample size	Experiments were performed as follows; tissue samples analyzed (n=4-35, as indicated in the figures), in vitro studies (N=2 for proliferation, adhesion, invasion and migration assay in duplicate/triplicate; for adhesion and proliferation assay, representative experiment is shown), RNA-seq (N=1-2, depending on cell type), ChIP-seq (n=1 for human tissues, 3 tissue/group).The number of animals per each group was determined based on our prior study using the same model (Marusyk et al., Nature 2014 and Hu et al. Cancer Cell 2008). We estimated that 5 animals per group would account for variability in tumor growth.
Data exclusions	N/A
Replication	Tumor growth assays were reproduced in multiple independent experiments.
Randomization	N/A
Blinding	Mouse experiment was repeated by technical assistants in the Lurie Imaging Center who were blinded to the identity of the samples. Similarly, some RNAseq and ChIPseq was performed by bioinformatician in the Center for Functional Cancer Epigenetics blinded to the identity of the samples.

Reporting for specific materials, systems and methods

We require information from authors about some types of materials, experimental systems and methods used in many studies. Here, indicate whether each material, system or method listed is relevant to your study. If you are not sure if a list item applies to your research, read the appropriate section before selecting a response.

Materials & experimental systems

n/a	Involved in the study	
☐	☒	Antibodies
☐	☒	Eukaryotic cell lines
☒	☐	Palaeontology
☐	☒	Animals and other organisms
☒	☐	Human research participants
☒	☐	Clinical data

Methods

n/a	Involved in the study	
☐	☒	ChIP-seq
☐	☒	Flow cytometry
☒	☐	MRI-based neuroimaging

Antibodies

Antibodies used	All antibodies used in this study are described in the text and detailed information provided in the methods.
Validation	Antibodies used for FACS and CYTOF staining were validated using cell lines with known expression of the marker, and the obtained percentages corresponded well to the expected values. IHC and IF antibodies were validated on cell-line derived xenografts with known expression of the marker. Immunoblot antibodies were validated on shRNA-expressing or overexpression cell lines.

Eukaryotic cell lines

Policy information about cell lines

Cell line source(s)	Cell line MCF10DCIS.com was obtained from Fred Miller, Karmanos Cancer Research Institute, Jurkat and DU4475 cell lines were from ATCC.
Authentication	MCF10DCIS.com, Jurkat and DU4475 cell lines were tested by STR and SNP array profiling by the DFCI Molecular Biology core facility, MCF10DCIS.com was also subject to whole genome sequencing.
Mycoplasma contamination	MCF10DCIS.com, Jurkat and DU4475 cell lines were routinely tested for mycoplasma and rodent pathogen contamination. No contamination was found at any time point.

Commonly misidentified lines (See ICLAC register)	*Name any commonly misidentified cell lines used in the study and provide a rationale for their use.*

Animals and other organisms

Policy information about studies involving animals; ARRIVE guidelines recommended for reporting animal research

Laboratory animals	4–6-weeks old female mice NCR-nude mice (Taconic) or NSG (Jackson Labs) were used following ACUC-approved protocols.
Wild animals	N/A
Field-collected samples	N/A
Ethics oversight	Animal experiments were performed by the Lurie Family Imaging Center following protocols approved by the Dana-Farber Cancer Institute Animal Care and Use Committee.

Note that full information on the approval of the study protocol must also be provided in the manuscript.

ChIP-seq

Data deposition

☒ Confirm that both raw and final processed data have been deposited in a public database such as GEO.

☒ Confirm that you have deposited or provided access to graph files (e.g. BED files) for the called peaks.

Data access links
May remain private before publication.

All raw genomic data was deposited to GEO under accession number GSE113909. Secure token for reviewer access while it remains in private status: cfyxmkkoxrmtrux

Files in database submission

MCF10DCIS_H3K27ac_peaks.filtered.narrowPeak
MCF10DCIS_p63_only.narrowPeak
MCF10DCIS-TetTCF7_H3K27ac.rep1_sorted_peaks.narrowPeak.bed
MCF10DCIS-TetTCF7_TCF7-HA_New.rep1_sorted_peaks.narrowPeak.bed
Myoep_N293_p63_peaks.narrowPeak
Myoep_N309_p63_peaks.narrowPeak
Myoep_N322_p63_peaks.narrowPeak
Myoep_N323_p63_peaks.narrowPeak
Myoep_N325_p63_peaks.narrowPeak
Myoep_N334_p63_peaks.narrowPeak
Myoep_N342_p63_peaks.narrowPeak
Myoep_N350_p63_peaks.narrowPeak
20141103-N309-p63-YS1437_S1_R1.fastq.gz
20160821-IdxF6-BJ039-Ft-p63-8-8-16-MD3366_S6_R1_001.fastq.gz
20160821-IdxG6-BJ040-FT-p63-8-8-16-MD3366_S7_R1_001.fastq.gz
20160229-BJ34-p63ChIP-IDX21-MD2819-2_S1_R1_001.fastq.gz
20160229-BJ36-p63ChIP-IDX23-MD2819-2_S3_R1_001.fastq.gz
20160229-BJ37-p63ChIP-IDX24-MD2819-2_S4_R1_001.fastq.gz
20160229-BJ35-p63ChIP-IDX22-MD2819-2_S2_R1_001.fastq.gz
20160821-IdxH6-BJ041-FT-p63-8-8-16-MD3366_S8_R1_001.fastq.gz
20141103-MCFDCIS-p63-YS1437_S5_R1.fastq.gz
20141110-Idx5-MCFDCIS-H3K27ac-MD1460_S5_R1.fastq.gz
20141110-Idx7-MCFDCIS-TetTCF7-H3K27ac-MD1460_S7_R1.fastq.gz
20141110-Idx3-MCFDCIS-Input-MD1460_S3_R1.fastq.gz
20180120_Dox_DSGPFA_inputKH5190_S4_R1_001.fastq.gz
Myoep_N250_H3K27ac_peaks_sorted.bed
Myoep_N293_H3K27ac_peaks_sorted.bed
Myoep_N274_H3K27ac_peaks_sorted.bed
Myoep_N296_H3K27ac_peaks_sorted.bed
20141103-N250-H3K27Ac-YS1437_S9_R1.fastq.gz
20141110-Idx11-N293-H3K27ac-MD1460_S8_R1.fastq.gz
20141103-N274-H3K27Ac-YS1437_S10_R1.fastq.gz
20140818-N296-H3K27Ac-YS1223_S10_R1.fastq.gz
YS8_N250_INPUT_08_08_13_GCCAAT_L002_R1_001.fastq.gz
BRCA1_201_TCF7.rep1_sorted_peaks.narrowPeak.bed
BRCA1_448_TCF7.rep1_sorted_peaks.narrowPeak.bed
BRCA2_156_TCF7.rep1_sorted_peaks.narrowPeak.bed
BRCA2_393_TCF7.rep1_sorted_peaks.narrowPeak.bed
Normal_309_TCF7.rep1_sorted_peaks.narrowPeak.bed
Normal_435_TCF7.rep1_sorted_peaks.narrowPeak.bed
TCF7_DU4415.rep1_sorted_peaks.narrowPeak.bed
20190122_309_input_KH6570_S1_R1_001.fastq.gz
20190122_393_input_KH6570_S9_R1_001.fastq.gz
20190122_393_TCF7_KH6570_S18_R1_001.fastq.gz

20190122_448_TCF7_KH6570_S15_R1_001.fastq.gz
20190122_309_TCF7_KH6570_S10_R1_001.fastq.gz
20190122_448_input_KH6570_S6_R1_001.fastq.gz
20190122_201_TCF7_KH6570_S13_R1_001.fastq.gz
20190122_435_TCF7_KH6570_S12_R1_001.fastq.gz
20190122_156_input_KH6570_S7_R1_001.fastq.gz
20190122_156_TCF7_KH6570_S16_R1_001.fastq.gz
20190122_201_input_KH6570_S4_R1_001.fastq.gz
20190122_435_input_KH6570_S3_R1_001.fastq.gz

Genome browser session
(e.g. UCSC)

Provide a link to an anonymized genome browser session for "Initial submission" and "Revised version" documents only, to enable peer review. Write "no longer applicable" for "Final submission" documents.

Methodology

Replicates

Describe the experimental replicates, specifying number, type and replicate agreement.

Sequencing depth

MCF10DCIS_H3K27ac_peaks.filtered.narrowPeak
MCF10DCIS_p63_only.narrowPeak
MCF10DCIS-TetTCF7_H3K27ac.rep1_sorted_peaks.narrowPeak.bed
MCF10DCIS-TetTCF7_TCF7-HA_New.rep1_sorted_peaks.narrowPeak.bed
Myoep_N293_p63_peaks.narrowPeak
Myoep_N309_p63_peaks.narrowPeak
Myoep_N322_p63_peaks.narrowPeak
Myoep_N323_p63_peaks.narrowPeak
Myoep_N325_p63_peaks.narrowPeak
Myoep_N334_p63_peaks.narrowPeak
Myoep_N342_p63_peaks.narrowPeak
Myoep_N350_p63_peaks.narrowPeak
20141103-N309-p63-YS1437_S1_R1.fastq.gz
20160821-IdxF6-BJ039-Ft-p63-8-8-16-MD3366_S6_R1_001.fastq.gz
20160821-IdxG6-BJ040-FT-p63-8-8-16-MD3366_S7_R1_001.fastq.gz
20160229-BJ34-p63ChIP-IDX21-MD2819-2_S1_R1_001.fastq.gz
20160229-BJ36-p63ChIP-IDX23-MD2819-2_S3_R1_001.fastq.gz
20160229-BJ37-p63ChIP-IDX24-MD2819-2_S4_R1_001.fastq.gz
20160229-BJ35-p63ChIP-IDX22-MD2819-2_S2_R1_001.fastq.gz
20160821-IdxH6-BJ041-FT-p63-8-8-16-MD3366_S8_R1_001.fastq.gz
20141103-MCFDCIS-p63-YS1437_S5_R1.fastq.gz
20141110-Idx5-MCFDCIS-H3K27ac-MD1460_S5_R1.fastq.gz
20141110-Idx7-MCFDCIS-TetTCF7-H3K27ac-MD1460_S7_R1.fastq.gz
20141110-Idx3-MCFDCIS-Input-MD1460_S3_R1.fastq.gz
20180120_Dox_DSGPFA_inputKH5190_S4_R1_001.fastq.gz
Myoep_N250_H3K27ac_peaks_sorted.bed
Myoep_N293_H3K27ac_peaks_sorted.bed
Myoep_N274_H3K27ac_peaks_sorted.bed
Myoep_N296_H3K27ac_peaks_sorted.bed
20141103-N250-H3K27Ac-YS1437_S9_R1.fastq.gz
20141110-Idx11-N293-H3K27ac-MD1460_S8_R1.fastq.gz
20141103-N274-H3K27Ac-YS1437_S10_R1.fastq.gz
20140818-N296-H3K27Ac-YS1223_S10_R1.fastq.gz
YS8_N250_INPUT_08_08_13_GCCAAT_L002_R1_001.fastq.gz
BRCA1_201_TCF7.rep1_sorted_peaks.narrowPeak.bed
BRCA1_448_TCF7.rep1_sorted_peaks.narrowPeak.bed
BRCA2_156_TCF7.rep1_sorted_peaks.narrowPeak.bed
BRCA2_393_TCF7.rep1_sorted_peaks.narrowPeak.bed
Normal_309_TCF7.rep1_sorted_peaks.narrowPeak.bed
Normal_435_TCF7.rep1_sorted_peaks.narrowPeak.bed
TCF7_DU4415.rep1_sorted_peaks.narrowPeak.bed
20190122_309_input_KH6570_S1_R1_001.fastq.gz
20190122_393_input_KH6570_S9_R1_001.fastq.gz
20190122_393_TCF7_KH6570_S18_R1_001.fastq.gz
20190122_448_TCF7_KH6570_S15_R1_001.fastq.gz
20190122_309_TCF7_KH6570_S10_R1_001.fastq.gz
20190122_448_input_KH6570_S6_R1_001.fastq.gz
20190122_201_TCF7_KH6570_S13_R1_001.fastq.gz
20190122_435_TCF7_KH6570_S12_R1_001.fastq.gz
20190122_156_input_KH6570_S7_R1_001.fastq.gz
20190122_156_TCF7_KH6570_S16_R1_001.fastq.gz
20190122_201_input_KH6570_S4_R1_001.fastq.gz
20190122_435_input_KH6570_S3_R1_001.fastq.gz

Antibodies

antibodies p63 (abcam, ab735), H3K27ac (abcam, ab4729), TCF7 (Sigma, WH0006932M1) and HA (abcam, ab9110)

Peak calling parameters

MACS2 (v 2.1.1.20160309) as a peak caller, with a q-value (FDR) threshold of 0.1

Data quality

QC passed in pipeline

Software

ChiLin pipeline 2.0.0 (Qin Q, Mei S, Wu Q, et al. ChiLin: a comprehensive ChIP-seq and DNase-seq quality control and analysis pipeline. BMC bioinformatics. 2016;17(1):404. doi:10.1186/s12859-016-1274-4.) is used for QC and preprocess of the ChIP-seq. We use Burrows-Wheeler Aligner (Li H. and Durbin R. (2009) Fast and accurate short read alignment with Burrows-Wheeler Transform. Bioinformatics, 25:1754-60. [PMID: 19451168]) as a read mapping tool, and Model-based Analysis of ChIP-Seq MACS2 (v 2.1.1.20160309) as a peak caller, with a q-value (FDR) threshold of 0.1. Based on a dynamic Poisson distribution MACS2 can effectively capture local biases in the genome sequence, allowing for more sensitive and robust prediction of binding sites. Unique read for a position for peak calling is used to reduce false positive peaks, statically significant peaks are finally selected by calculated false discovery rate of reported peaks.

Following QC methods have applied to the ChIP-seq data. i) sequence quality QC, we calculates these scores using the FastQC software[4]. A good sequence quality score is ≥ 25 ; ii) PCR Bottleneck Coefficient - PBC score >= 0.90; iii) percentage overlap with known DHSs derived from the ENCODE Project (the minimum required was 70%); iv)peak conservations; v) number of total peaks (the minimum required was 1,000).

Flow Cytometry

Plots

Confirm that:

☒ The axis labels state the marker and fluorochrome used (e.g. CD4-FITC).

☒ The axis scales are clearly visible. Include numbers along axes only for bottom left plot of group (a 'group' is an analysis of identical markers).

☒ All plots are contour plots with outliers or pseudocolor plots.

☒ A numerical value for number of cells or percentage (with statistics) is provided.

Methodology

Sample preparation

Single-cell suspensions of human breast epithelial cells were obtained as described in Shipitsin, 2007, Cancer Cell.
Samples for BrdU staining have been pulsed with BrdU (10µM) for 1h and subsequently stained with the FITC BrdU Flow Kit (BD Biosciences) according to the manufacturer's instruction.

Instrument

M Fortessa and M Aria II SORP UV were used for data collection.

Software

BD FACSDiva 8.0.1 was used for data collection and FlowJo v10 was used for data analysis. Cytobank was used for data analysis of BrdU assay.

Cell population abundance

We collected at least 100,000 cells in each cell fraction. For BrdU analysis, data from 10,000 single cells has been collected and analyzed.

Gating strategy

Gating strategy used for the quantification of CD10+CD44+ and CD10+CD44- cells. Single cell suspension derived from organoids were stained with viability dye-pacific blue, CD24-APC, CD44-PE, and CD10-FITC. Each single-color control was run in parallel to ensure proper compensation. Cells were gated using forward scatter (FSC) and side scatter (SSC) and then gated for the pacific blue negative viable cells. Next, CD24-APC+ cells were gated and sorted for other studies. The remaining CD24-APC negative cells were gated for CD10-FITC+CD44-PE+ and CD10-FITC+CD44-PE- cells and sorted.
For cell cycle analysis using BrDU-labelled cells, single cells were gated based on 7-AAD-H/7-AAD-A. Singlets were subsequently gated for S-phase (FITC-BrdU positive, G1-phase (single DNA (7-AAD)-content) and G2/M-phase (double (7-AAD)-content)).

☒ Tick this box to confirm that a figure exemplifying the gating strategy is provided in the Supplementary Information.
